# Exosomes as nanocarriers for systemic delivery of the *Helicobacter pylori* virulence factor CagA

**DOI:** 10.1038/srep18346

**Published:** 2016-01-07

**Authors:** Asako Shimoda, Koji Ueda, Shin Nishiumi, Naoko Murata-Kamiya, Sada-atsu Mukai, Shin-ichi Sawada, Takeshi Azuma, Masanori Hatakeyama, Kazunari Akiyoshi

**Affiliations:** 1 Department of Polymer Chemistry, Graduate School of Engineering, Kyoto University, Katsura, Nishikyo-ku, Kyoto 615-8510, Japan; 2 Japan Science and Technology Agency (JST), The Exploratory Research for Advanced Technology (ERATO), Bio-nanotransporter Project, Katsura Int’tech Center, Katsura, Nishikyo-ku, Kyoto 615-8530, Japan; 3 Division of Biosciences, Functional Proteomics Center, Graduate School of Frontier Sciences, the University of Tokyo, CREST hall 1F, Institute of Medical Science, 4-6-1 Shirokanedai, Minato-ku, Tokyo 108-8639, Japan; 4 Division of Gastroenterology, Department of Internal Medicine, Kobe University Graduate School of Medicine, 7-5-1, Kusunoki-cho, Chu-o-ku, Kobe, Hyogo 650-0017, Japan; 5 Department of Microbiology, Graduate School of Medicine, the University of Tokyo, 7-3-1 Hongo, Bunkyo-Ku, Tokyo 113-0033, Japan

## Abstract

CagA, encoded by cytotoxin-associated gene A (*cagA*), is a major virulence factor of *Helicobacter pylori*, a gastric pathogen involved in the development of upper gastrointestinal diseases. Infection with *cagA*-positive *H. pylori* may also be associated with diseases outside the stomach, although the mechanisms through which *H. pylori* infection promotes extragastric diseases remain unknown. Here, we report that CagA is present in serum-derived extracellular vesicles, known as exosomes, in patients infected with *cagA*-positive *H. pylori* (n = 4). We also found that gastric epithelial cells inducibly expressing CagA secrete exosomes containing CagA. Addition of purified CagA-containing exosomes to gastric epithelial cells induced an elongated cell shape, indicating that the exosomes deliver functional CagA into cells. These findings indicated that exosomes secreted from CagA-expressing gastric epithelial cells may enter into circulation, delivering CagA to distant organs and tissues. Thus, CagA-containing exosomes may be involved in the development of extragastric disorders associated with *cagA*-positive *H. pylori* infection.

*Helicobacter pylori* is a gram-negative bacterium that colonises the human stomach. Nearly half of the world population has been estimated to be carriers of *H. pylori*, and infection with *H. pylori* is associated with the pathogenesis of gastric disorders, such as atrophic gastritis, peptic ulcers, and gastric cancer^1^,^2^,^3^. Among reported *H. pylori* virulence factors, much attention has been focused on CagA, a protein encoded by *cytotoxin associated gene A* (*cagA*), because of its strong association with severe gastric lesions, particularly gastric cancer^4^,^5^. CagA is a bacterial effector protein that is injected into gastric epithelial cells via the type IV secretion system (T4SS). Inside host epithelial cells, CagA undergoes tyrosine phosphorylation by Src family kinases (SFKs) and acts as a pathogenic scaffold that promiscuously perturbs signalling pathways regulating cell growth, motility, and polarity, thereby promoting neoplastic transformation of cells^6^,^7^,^8^,^9^,^10^,^11^,^12^. Indeed, transgenic mice systemically expressing the bacterial *cagA* gene exhibit gastric epithelial hyperplasia, gastrointestinal carcinomas, and B cell lymphomas, indicating that CagA acts as an oncoprotein in mammals^13^.

Recent studies have also suggested that *H. pylori* infection is involved in the development of diseases outside the stomach, including cardiovascular diseases, haematologic diseases, diabetes mellitus, idiopathic parkinsonism, and others^14^,^15^,^16^,^17^,^18^. Additionally, *H. pylori* or other *Helicobacter* species have been detected in atherosclerotic plaques from patients; several mechanisms have been proposed, including direct effects of the bacterium on the vascular wall to induce endothelial dysfunction, indirect effects including the promotion of systemic inflammation and production of inflammatory mediators, and induction of molecular mimicry by the production of cross-reactive antibodies^19^,^20^. However, the specific mechanisms mediating the extragastric effects of *H. pylori* remain unclear. In particular, infection with *H. pylori cagA*-positive strains has been shown to be associated with coronary heart diseases^21^,^22^,^23^ and ischemic stroke^24^,^25^,^26^. Preeclampsia, a pregnancy-associated disorder characterised by hypertension and proteinuria, is another vascular disease related to *cagA*-positive *H. pylori* infection^27^,^28^. These studies indicate that CagA may also contribute to the development of extragastric diseases^29^.

Many types of cells are known to release extracellular vesicles (EVs) with unique biophysical and biochemical properties^30^,^31^. These vesicles are classified based on their biogenesis; vesicles formed by exocytosis of multivesicular bodies are referred as exosomes (with diameters ranging from 30 to 200 nm), while vesicles budded directly from the plasma membrane are referred to as microvesicles (with diameters ranging from 100 to 1000 nm)^32^. EVs are found in various biological fluids, including blood, urine, saliva, and breast milk, and they have been shown to play an important role in cell-to-cell communication through transport of informative constituents, including proteins, lipids, and microRNAs (miRNAs)^33^,^34^. Many exosomal miRNAs have been identified, and their sorting is modulated in a cell-dependent manner. For example, exosomes containing miRNAs released from cancer cells are involved in tumorigenesis and metastasis and have been shown to act as cancer biomarkers^35^. Recent studies have also demonstrated the role of EVs in the transfer of proteins during infection, including prion protein (PrP) in neurodegenerative disease^36^, human immunodeficiency virus (HIV)-related proteins^37^, and human T-cell leukaemia virus type-1 (HTLV-1) proteins^38^. However, functions of exosomes as nanocarriers of pathogen-associated molecules during the development of various diseases are not well understood.

In this study, we aimed to elucidate the mechanisms through which CagA induces extragastric lesions in individuals infected with *cagA*-positive *H. pylori*. We found that exosomes containing CagA were released from *H. pylori* CagA-expressing cells and were detectable in the blood circulation, suggesting that CagA-containing exosomes could mediate the development of multiple extragastric diseases.

## Results

### Detection of CagA in serum exosomes isolated from *cagA*-expressing *H. pylori*-positive patients with gastric cancer

**Figure 1:**
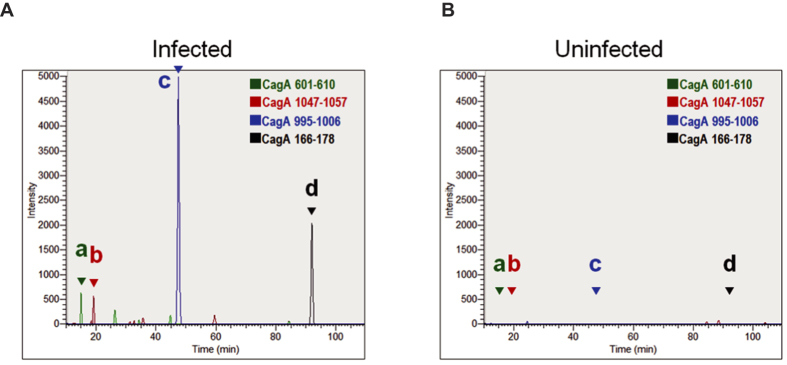
Mass spectrometric identification of exosomal CagA protein in *H. pylori*-infected human serum. Exosomes were isolated from *H. pylori*-infected. (**A**) or uninfected (**B**) human serum and analysed by LC-MS/MS. Extracted ion chromatograms (XICs) of CagA-derived peptides are shown. The targeted mass-to-charge ratios (*m/z* values) were 584.2863 (**a**), 646.3060 (**b**), 406.5489 (**c**), or 708.9030 (**d**), respectively. Peak IDs in a–d correspond to those in Table 1.

Serum-derived exosomes were collected from *cagA*-positive *H. pylori*-infected patients with gastric cancer (the sample was prepared by mixing sera from four patients) and from *H. pylori*-uninfected healthy donor (n = 1) by ultracentrifugation. Transmission electron microscopy (TEM) and nanoparticle tracking analysis (NTA) demonstrated the presence of round-shaped vesicles measuring 20–150 nm (Supplementary Figure S1). To investigate whether CagA was present in the exosomes from *cagA*-positive *H. pylori*-infected patients, exosomes were analysed by liquid chromatography-tandem mass spectrometry (LC-MS/MS). Detection of known exosomal surface antigens, including CD9, CD63 (see Supplementary Table S1 and Supplementary Figure S2), CD81, and CD82^30^, indicated that exosomes were effectively enriched by the ultracentrifugal procedure (Supplementary Tables S2 and S3). From SEQUEST database search analysis, four CagA-derived tryptic peptides were significantly identified with a false discovery rate (FDR) of less than 1% (Table 1 and Supplementary Figure S3); the amino acid sequences of these peptides were identical to *H. pylori* Japanese strain F32. The extracted ion chromatograms of four peptides (CagA 166–178, 601–610, 995–1006, and 1047–1057) acquired from *H. pylori*-infected or -uninfected individuals are shown in Fig. 1A,B, respectively. All four peptides were specifically detected in serum exosomes from *H. pylori-*infected patients with gastric cancer but not from uninfected individuals. Other virulence factors from *H. pylori*, including Gamma-glutamyltransferase (GGT) and VacA, were not detected in serum-derived exosomes. These data revealed that circulating serum exosomes in *H. pylori*-infected patients contained CagA, suggesting that CagA could be delivered to nongastrointestinal compartments via circulating exosomes.

### Isolation and characterisation of exosomes from CagA-expressing gastric epithelial cells

**Figure 2:**
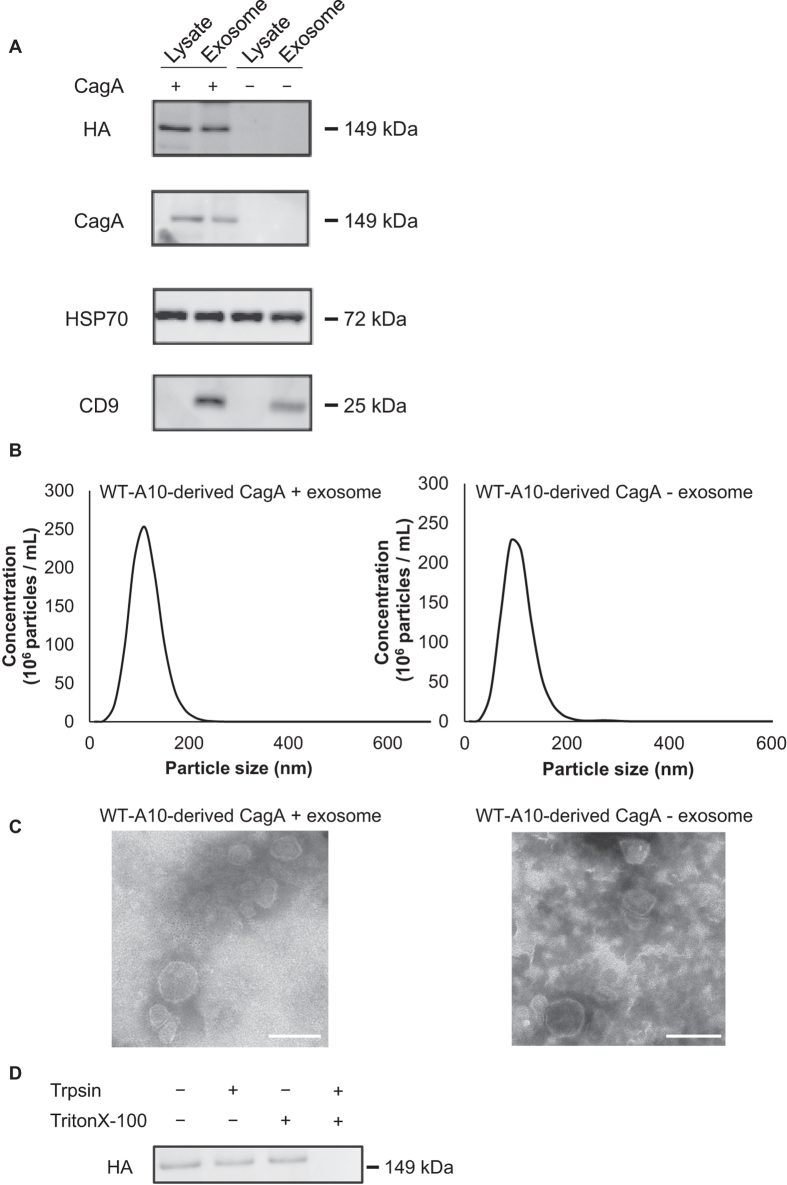
Characterisation of WT-A10 cell-derived exosomes. (**A**) WT-A10 cell lysates and exosomes were analysed by western blotting using antibodies against HA, CagA, and exosomal markers (CD9 and HSP70). Inducible expression of HA-tagged CagA in WT-A10 cells was regulated by treatment with or without doxycycline (Dox). (**B**) The size distribution of CagA-positive exosomes (left) and CagA-negative exosomes (right) obtained by NTA. The data represent the mean ± SD (n = 5). (**C**) Morphologies of CagA-positive exosomes (left) and CagA-negative exosomes (right) were observed by TEM. Scale bar = 100 nm. (**D**) CagA protein was found inside the exosome. CagA-positive exosomes were incubated with trypsin in the absence or presence of 0.2% TritonX-100 at 37 °C for 5 min. The samples were separated by SDS-PAGE and analysed by western blotting using antibodies against HA.

To investigate the mechanism underlying the generation of CagA-positive exosomes and to examine the biological functions of CagA-containing exosomes, we used WT-A10 gastric epithelial cells, which can be induced to express wild-type CagA via a tetracycline-regulated Tet-off system, which mimics CagA injection by *H. pylori* into the host cells^8^, and the level of the CagA protein inducibly expressed in WT-A10 cells is roughly comparable to that expressed in AGS cells co-cultured with *H. pylori*^11^. After culturing WT-A10 cells in the absence or presence of doxycycline (Dox), a tetracycline analogue, exosomes were collected from culture supernatants using differential centrifugation and analysed by sodium dodecyl sulphate polyacrylamide gel electrophoresis (SDS-PAGE) and western blotting. CagA was specifically detected in cell lysates and exosomes prepared from CagA-expressing cells (Dox–, CagA +; Fig. 2A). We also confirmed the presence of the exosome markers CD9 and heat-shock protein 70 (HSP70)^39^. These marker proteins were detected in both CagA-expressing exosomes and exosomes not containing CagA. Next, the morphologies and sizes of exosomes were evaluated using TEM and NTA (Fig. 2B,C). TEM images showed round-shaped vesicles with lipid bilayers. The average diameters of exosomes were around 120 nm, as shown by NTA analysis.

To assess the localisation of CagA in the exosomes, trypsin digestion assays were performed with or without detergent (TritonX-100), followed by western blot analysis^40^,^41^. When the exosomes were treated with trypsin in the absence of TritonX-100, CagA was protected from trypsin-dependent enzymatic degradation. In contrast, in the presence of both trypsin and TritonX-100, CagA was completely degraded (Fig. 2D and Supplementary Figure S4). These results indicated that CagA was located inside the exosomes.

### Induction of CagA-dependent morphological changes by CagA-containing exosomes

**Figure 3:**
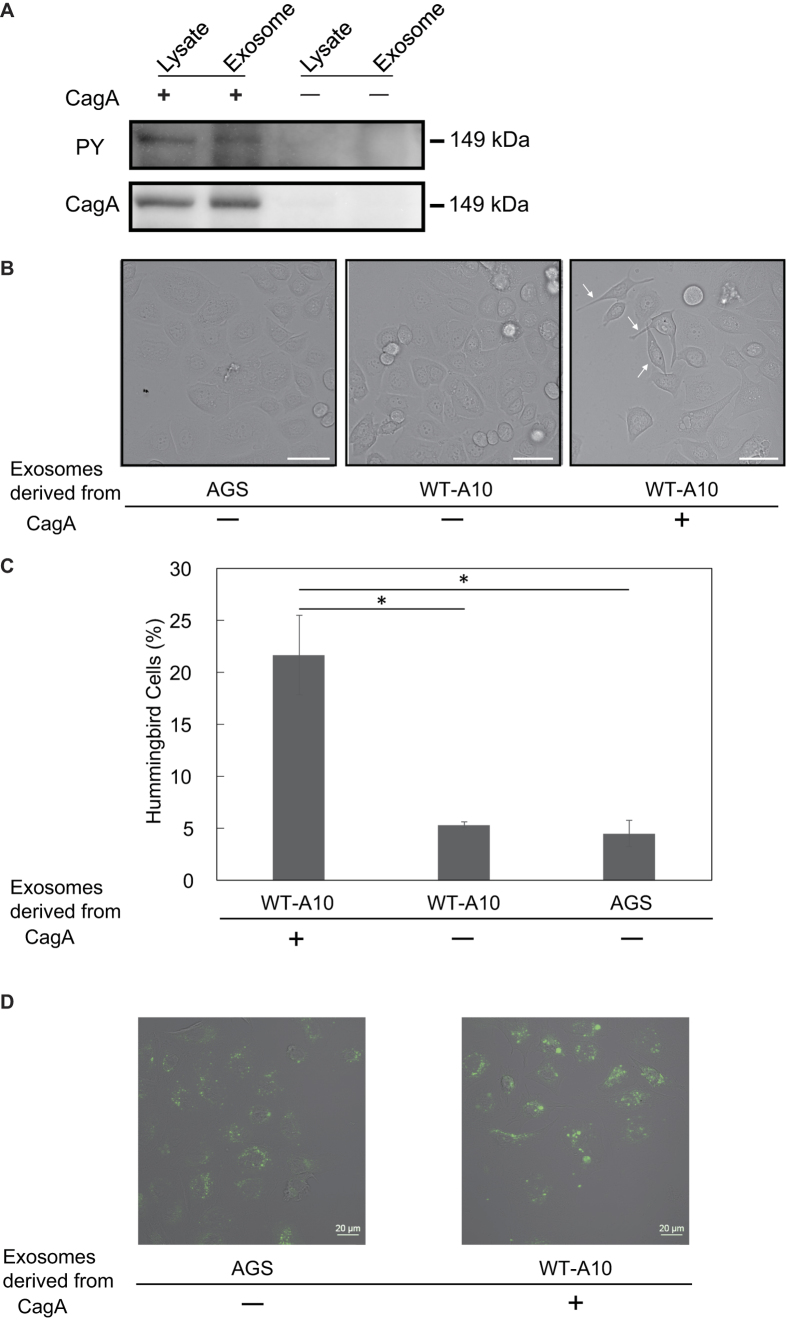
CagA-positive exosomes induced morphological changes in AGS cells. (**A**) Detection of tyrosine-phosphorylated CagA in whole cell lysates and exosomes by western blotting using an anti-phosphotyrosine antibody. The same membrane blot was stripped and reprobed with anti-CagA antibody. (**B**) AGS-derived exosomes, WT-A10-derived CagA-negative exosomes, and WT-A10-derived CagA-positive exosomes were added to AGS cells (5 μg protein/ 3.5 × 10^4^ cells), and cell morphologies were observed by light microscopy. Arrows indicate the hummingbird phenotype. Scale bar = 50 μm. (**C**) The percentages of hummingbird cells induced by CagA-negative and CagA-positive exosomes were calculated. Error bars indicate the mean ± SD (n = 3); *p < 0.001, Student’s t-test. (**D**) Uptake of CagA-negative and CagA-positive exosomes. These exosomes were labelled with PKH67 dye as described in the Methods. PKH67-labeled exosomes were added to AGS cells (5 μg protein/3.5 × 10^4^ cells). Cells were visualised using a confocal laser scanning microscope.

In host gastric epithelial cells, CagA undergoes tyrosine phosphorylation by SFKs. Since tyrosine phosphorylation activates the ability of CagA to induce an elongated cell-shape, known as the hummingbird phenotype^11^, we hypothesised that CagA protein in exosomes released from CagA-expressing gastric epithelial cells may also contain the tyrosine-phosphorylated form of CagA. To test this idea, cell lysates and exosomes prepared from CagA-expressing cells or cells not expressing CagA were separated by SDS-PAGE and subjected to western blot analysis using an anti-phosphotyrosine antibody (anti-PY). The results revealed that tyrosine-phosphorylated CagA was present in both cell lysates and exosomes prepared from CagA-expressing cells (Fig. 3A and Supplementary Figure S5). Accordingly, exosomes released from CagA-expressing gastric epithelial cells contained tyrosine-phosphorylated CagA.

Tyrosine-phosphorylated CagA specifically induces a highly elongated cell-shape known as the hummingbird phenotype in gastric epithelial cells^11^. Thus, we next investigated whether treatment of gastric epithelial cells with CagA-containing exosomes could induce morphological changes in the cells upon delivery of tyrosine-phosphorylated CagA. For this experiment, CagA-expressing exosomes and exosomes not containing CagA, prepared from induced WT-A10 cells expressing CagA and uninduced WT-A10 cells or AGS cells not expressing CagA, were added to cultures of AGS cells (5 μg protein/3.5 × 10^4^ cells). At 24 h after exosome treatment, the morphology of AGS cells was evaluated by microscopy. When AGS cells were treated with CagA-negative exosomes, no overt morphological changes were observed (Fig. 3B). In contrast, treatment of AGS cells with CagA-positive exosomes elicited the hummingbird phenotype in approximately 20% of cells (Fig. 3C). These results indicated the exosomes delivered tyrosine-phosphorylated CagA into AGS cells and subsequently induced morphological changes in the cells. Practically, it is impossible to determine the ratio of the phosphorylation of CagA proteins in exosomes from the intensity of the bands by Western blot analysis because the anti-PY antibody and the anti-CagA antibody have different affinities to the CagA proteins. Therefore, CagA proteins within exosomes isolated from WT-A10 cells may exist as a mixture of phosphorylated-CagA and non-phosphorylated CagA. Even so, it may be possible for non-phosphorylated CagA to be phosphorylated in recipient cells and to induce the hummingbird phenotype. Consistent with this, internalisation of PKH67-labelled exosome in AGS cells was observed by confocal laser-scanning microscopy. Thus, exosomes were efficiently internalised into AGS cells regardless of the expression of CagA protein (Fig. 3D).

## Discussion

In this study, we investigated the mechanisms through which CagA can induce extragastric lesions upon *H. pylori* infection. We found that exosomes containing CagA were detectable in the blood of individuals infected with *cagA*-positive *H. pylori*. We also found that the CagA-containing exosomes were released from gastric epithelial cells expressing CagA. Collectively, these findings suggested that *H. pylori* CagA-containing exosomes could facilitate the development of multiple extragastric diseases.

Infection with *H. pylori*, particularly virulent *cagA*-positive strains, is causally associated with the development of severe gastric diseases, such as peptic ulcerations and gastric cancer. *H. pylori cagA*-positive strains specifically colonise the human stomach, where they deliver the *cagA*-encoded CagA protein into gastric epithelial cells via type IV secretion. We assumed that *H. pylori*-delivered CagA is released from the host gastric epithelial cells as a component of exosomes, which enter into systemic circulation and thereby deliver CagA to remote organs and/or tissues through the blood. Tyrosine-phosphorylated CagA is the active form of CagA and has been shown to impair physiological cellular functions by acting as a pathogenic scaffold that perturbs multiple intracellular signalling pathways^4^,^11^. A previous study demonstrated that the biological half-life of unphosphorylated or tyrosine-phosphorylated CagA is relatively short (approximately 200 min) in gastric epithelial cells^42^. On the other hand, our finding suggested that CagA secreted from gastric epithelial cells existed stably in the tyrosine-phosphorylated form within exosomes.

Recent epidemiological studies have also suggested correlations between *H. pylori* infection and several nongastrointestinal diseases, most notably cardiovascular diseases^43^. Anti-CagA antibodies are suspected to cross-react with self-antigens exposed on the surface and/or present in the cytoplasm of endothelial cells, thereby triggering inflammation and deteriorating vascular lesions underlying coronary heart diseases, cerebral stroke, and preeclampsia^44^,^45^. Chronic gastritis caused by *cagA*-positive *H. pylori* also induces a mild but systemic inflammation status via increased levels of circulating pro-inflammatory cytokines^46^, which further accelerates the development of cardiovascular diseases. Our study suggested that CagA may contribute to the development of vascular lesions via delivery by exosomes, providing a novel mechanism to explain the extragastric effects of *H. pylori* infection.

GGT, another virulence factor in *H. pylori*, induces apoptosis in gastric epithelial cells^47^. GGT in *H. suis*, a relative of *H. pylori* that may also cause gastric lesions, is secreted in the form of exosome-like vesicles (outer membrane vesicles [OMVs]), which can translocate across the epithelial layers and deliver the enzyme to mucosal lymphocytes, thereby inhibiting their proliferation^48^. Thus, OMVs can be regarded as pathogenic carriers from the bacteria to the host cells. Vacuolating cytotoxin A (VacA), yet another important virulence factor in *H. pylori*, is released in both free form and membrane-associated form and enters epithelial cells via endocytosis^49^. Although *H. pylori* can release OMVs, which contain bacterial components including CagA, in the stomach lumen where the stomach pathogen colonizes, the presence of OMVs in the host (human) blood has not been reported so far. Furthermore, the biogenesis and components of OMVs are different from these of human cells-derived exosomes^50^. We found for the first time that CagA-containing exosomes are detectable in the blood of individuals infected with *cagA*-positive *H. pylori* in the stomach and at the same time demonstrated that exosomes released from from gastric epithelial cells inducibly expressing CagA were effectively internalised by epithelial cells and induced an elongated cell shape, indicating that the exosomes delivered functional CagA into cells. The functional role of exosomes as a transporter of various substances has been recently described^51^. The exosomes are internalised into various cells by endocytosis or membrane fusion^52^. CagA-containing exosomes circulating within blood vessels may adhere to the endothelial monolayer and deliver the CagA protein into endothelial cells. Since CagA stimulates the nuclear factor kappaB (NF-κB) signalling pathway in a cell-autonomous manner^53^,^54^ hyperactivated NF-κB in CagA-delivered endothelial cells may promote atherosclerosis, a chronic inflammatory condition leading to endothelial cell dysfunction and eventually plaque formation and evolution, thereby predisposing individuals to coronary heart diseases, such as angina pectoris and myocardial infarction^55^. CagA-containing exosomes may also directly fuse with atherosclerotic plaques. This in turn enables exposure of CagA on the plaque surface and allows recognition by anti-CagA antibodies, thereby triggering immune reactions in the atherosclerotic lesion. In the case of idiopathic thrombocytopenic purpura (ITP), anti-CagA antibodies have been reported to cross-react with a 55-kDa platelet protein, suggesting molecular mimicry mechanisms underlying the development of ITP^56^. However, because the molecular nature of the protein recognised by anti-CagA antibodies has not been elucidated, it is possible that the 55-kDa protein is a CagA fragment delivered into platelets via exosomes. Because CagA is a bacterial oncoprotein^57^, exosome-mediated CagA delivery may also be involved in the development of neoplasias outside the stomach. In fact, epidemiological studies suggested that infection with *H. pylori cagA*-positive strains increases the risk of colorectal cancer^58^ and pancreatic cancer^59^. Thus, further studies are required to determine the potential role of CagA-containing exosomes in the enhancement of oncogenesis other than gastric cancer.

In conclusion, our study revealed an unprecedented mechanism for the delivery of microbial virulence factors to sites distant from the primary infection site, which may provoke more systemic clinical effects or symptoms that are seemingly unrelated to the primary infection. Further studies are needed to determine whether such a mechanism may also be involved in chronic virus infection or colonisation of commensal bacteria.

## Methods

### Serum samples

This study was approved by the ethics committee of Kobe University Graduate School of Medicine, and human samples were used in accordance with the guidelines of Kobe University Hospital. Blood samples were collected from four patients with gastric cancer, who were diagnosed at Kobe University Hospital. Written informed consent was obtained from all participants. Serum samples were prepared using a standard venous blood sampling protocol. The collected blood was centrifuged at 3,000 × *g* for 10 min at 4 °C, and the serum was then transferred to a clean tube, followed by storage at −80 °C until use. Serum anti-*H. pylori* IgG levels of greater than 10 U/mL were judged as positive for *H. pylori* infection; the four patients with gastric cancer in this study were positive for *H. pylori* infection. To isolate a sufficient amount of exosomes from serum, serum samples obtained from the four patients with gastric cancer were mixed, and exosomes were then isolated from the combined serum sample. Serum from a healthy volunteer was also collected as described above, and anti-*H. pylori* IgG levels in the serum from this healthy volunteer were below detection limit; this serum preparation was used as the control.

### Cell cultures

WT-A10 cells, inductively expressing haemagglutinin (HA)-tagged wild-type CagA, were maintained as described previously^8^. WT-A10 cells were cultured in RPMI1640 medium supplemented with 10% heat-inactivated foetal bovine serum (FBS; depleted of exosomes), 10 mM HEPES, 1 mM sodium pyruvate, 0.1 mM nonessential amino acids, 2 mM l-glutamine, penicillin G sodium, streptomycin sulphate (1 × ), 500 μg/mL G418, 100 μg/mL hygromycin B, and 1 μg/mL doxycycline (Dox) at 37 °C in an atmosphere containing 5% CO_2_. Exosome-depleted FBS was prepared by ultracentrifugation at 120,000 × *g* for 5 h at 4 °C using a 50.2 Ti rotor (Beckman Coulter). AGS cells were cultured in RPMI1640 medium supplemented with 10% heat-inactivated FBS, 10 mM HEPES, 1 mM sodium pyruvate, 0.1 mM nonessential amino acids, penicillin G sodium, streptomycin sulphate (1×), 2.2 g/L sodium bicarbonate solution, and 50 μM 2-mercaptoethanol.

### Isolation of exosomes from cell culture supernatants

CagA expression was controlled by the presence or absence of Dox. WT-A10 cells expressing CagA were cultured without Dox for 96 h. Exosomes derived from WT-A10 cells were isolated by differential centrifugation^60^. Briefly, supernatants were centrifuged at 300 × *g* for 10 min, 2,000 × *g* for 10 min, and 10,000 × *g* for 30 min to remove dead cells and cell debris. After filtration through a 0.22-μm filter (Stericup, Millipore Corp., Bedford, MA, USA), the resulting supernatants were centrifuged at 120,000 × *g* for 100 min. Exosome pellets were washed with phosphate-buffered saline (PBS) and resuspended in a small volume of PBS for further analysis. Protein concentrations were determined using a Micro BCA assay kit (Pierce, Rockford, IL, USA). Exosomes derived from AGS cells, which did not express CagA, were also isolated by the same procedure.

### Nanoparticle tracking analysis (NTA)

The size distribution of exosomes was determined using a NanoSight LM10 (NanoSight, Amesbury, UK) with a blue laser. The exosome solution was diluted to about 10^8^–10^9^ particles/mL for analysis.

### Western blotting

CagA protein and exosome markers (CD9 and HSP70) were detected by western blotting. Equal amounts of cell lysates and exosomes were separated by 7.5% or 12.5% SDS-PAGE and transferred to polyvinylidene difluoride membranes (ATTO Co., Ltd., Japan). After blocking with 0.5% skim milk or Blocking One-P (Nacalai Tesque Inc., Kyoto, Japan) in TBST (20 mM Tris, 500 mM NaCl, pH 7.4, 0.05% Tween 20), membranes were blotted with the following primary antibodies: anti-HA antibodies (3F10; Roche), anti-CagA antibodies (AUSTRAL Biologicals), anti-phosphotyrosine antibodies (ab10321 [PY20]; Abcam), anti-CD9 antibodies (ab92726; Abcam), and anti-HSP70 antibodies (ab5439; Abcam). The membranes were then incubated with horseradish peroxidase (HRP)-conjugated secondary antibodies and ECL Western Blotting Detection Reagents (GE Healthcare). The bands were visualised using LAS-4000 (GE-Healthcare). For detection of tyrosine-phosphorylated CagA, the blot was first probed with anti-phosphotyrosine antibodies, stripped with EzReprobe reagent (ATTO Co., Ltd., Japan), and then reprobed with anti-CagA antibodies.

### Transmission electron microscopy (TEM)

Exosomes were placed on 100-mesh formvar-coated copper grids pretreated with 1% Alcian blue for 5 min. After rinsing with distilled water, exosomes in 2% paraformaldehyde (PFA) were absorbed onto grids for 10 min. Samples were stained with 2.5% phosphotungstic acid and examined using an HT7700-TEM (Hitachi, Japan) at an accelerating voltage of 100 kV.

### CagA localisation on exosomes

To determine CagA localisation on exosomes, exosomes were incubated with trypsin (16.6 μg/1 μg exosomes) in the absence or presence of 0.2% TritonX-100 at 37 °C for 5 min. The samples were then immediately mixed with SDS sample buffer, heated at 95 °C for 5 min, and analysed by SDS-PAGE and western blotting.

### Uptake of PKH67-labeled exosomes into AGS cells

Exosomes were labelled with a PKH67 Green Fluorescent Cell Linker Kit for General Cell Membrane Labelling (Sigma-Aldrich, St. Louis, MO, USA) according to the manufacturer’s protocol. Briefly, exosome solution in 1 mL of Diluent C was mixed with PKH67 dye solution in Diluent C and then incubated for 5 min. The reaction was stopped by adding an equal volume of 1% BSA. PKH67-labelled exosomes were isolated by ultracentrifugation and resuspended in PBS. To investigate the localisation of exosomes in AGS cells, PKH67-labelled CagA-positive exosomes or CagA-negative exosomes (5 μg/well) were incubated with AGS cells for 24 h. The cells were washed three times with PBS and then fixed in 4% paraformaldehyde. Cells were washed three times with PBS, mounted with ProLong antifade reagent (Invitrogen), and observed using a confocal laser scanning microscope LSM780 (Carl Zeiss, Germany).

### Changes in the morphology of AGS cells after exposure to CagA-expressing exosomes

AGS cells (2.1 × 10^4^ cells/well) were seeded into CELLview glass-bottomed dishes (Greiner BIO-ONE, Frickenhausen, Germany) for 24 h. Five micrograms of CagA-positive or CagA-negative exosomes were added to the cells and incubated for 24 h. Changes in AGS cell morphology (i.e., the hummingbird phenotype) were examined by microscopy. The hummingbird phenotype was defined as cells in which the ratio of the longest to shortest diameters was greater than 2.5-fold. Two hundred cells from each sample were counted for each of three independent experiments and analysed using NIH Image J (NIH, Bethesda, MD, USA).

### Isolation of exosomes from serum

Isolation of exosomes from serum was carried out using a method similar to that described above. Briefly, serum was diluted with PBS before centrifugation at 300, 2000, and 10,000 × *g*. The supernatant was further ultracentrifuged at 120,000 × *g*. Exosome pellets were washed with PBS and resuspended in a small volume of PBS for further analysis. The sizes and morphologies of serum-derived exosomes were analysed by NTA and TEM, respectively.

### LC-MS/MS analysis of serum-derived exosomes

Exosomal proteins were lysed in Laemmli sample buffer and reduced with 10 mM Tris (2-carboxyethyl) phosphine (Sigma-Aldrich) for 30 min at 37 °C, followed by alkylation with 50 mM iodoacetamide (Sigma-Aldrich) for 45 min in the dark at 25 °C. Proteins were separated on SDS-PAGE and stained with CBB. After desalting and drying excised gel bands, proteins were digested with Trypsin GOLD (Promega, Madison, WI, USA) at 37 °C for 16 h. The resulting peptides were extracted and analysed using an LTQ-Orbitrap-Velos mass spectrometer (Thermo Fisher Scientific) combined with an UltiMate 3000 RSLC nano-flow HPLC system (DIONEX Corporation). Protein identification analysis was performed using a SEQUEST database search with Proteome Discoverer 1.4 software (Thermo Fischer Scientific). The tandem mass spectrometry (MS/MS) spectra were searched against the *Homo sapiens* + *Helicobacter pylori* protein database in SwissProt. A false discovery rate of 1% and peptide rank of 1 were set for peptide identification filters. Extracted ion chromatograms were depicted with mass tolerance at 1 ppm.

### Statistical analysis

Statistical tests were performed with two-tailed Student’s t-tests. Differences with *P*-values of less than 0.001 were considered statistically significant.

## Additional Information

**How to cite this article**: Shimoda, A. *et al.* Exosomes as nanocarriers for systemic delivery of the *Helicobacter pylori* virulence factor CagA. *Sci. Rep.* **6**, 18346; doi: 10.1038/srep18346 (2016).

## Supplementary Material

Supplementary Information

## Figures and Tables

**Table 1:** CagA protein identified in *H. pylori*-infected human serum exosomes by LC-MS/MS.

PeakID^1^	*m/z*^2^ (Da)	Charge	RT^3^(min)	UniProtEntry	Protein description	Amino acidnumber	Sequence	XCorr^4^	SpScore^5^
a	584.2863	2	15.1	E6NP29	cag pathogenicity island protein	601–610	NTGNYDEVKK	2.11	240
b	646.3060	2	19.3	E6NP29	cag pathogenicity island protein	1047–1057	LDNYATNSHTR	2.12	627
c	406.5489	3	47.8	E6NP29	cag pathogenicity island protein	995–1006	IGDLSQAVSEAK	1.42	243
d	708.9030	2	92.1	E6NP29	cag pathogenicity island protein	166–178	QSFAGIIIGNQIR	3.88	1041

^1^IDs correspond to peaks in Figure 1.

^2^Mass-to-charge ratio.

^3^Retention time of nano-HPLC.

^4^Cross correlation score by SEQUEST search.

^5^Identity threshold score by SEQUEST search.
