# Supplementary material for: Exosomes as nanocarriers for systemic delivery of the Helicobacter pylori virulence factor CagA
Source: Sci Rep. 2016 Jan 7;6:18346. doi: 10.1038/srep18346 (PMC4703974; doi:10.1038/srep18346)
Supplement: Supplementary Information [file srep18346-s1.doc]

**Supplementary information　Figure S1-5, Table S1-3**

Exosomes as nanocarriers for systemic delivery of the *Helicobacter pylori* virulence factor CagA

Asako Shimoda^1, 2^, Koji Ueda^3^, Shin Nishiumi^4^, Naoko Murata-Kamiya^5^, Sada-atsu Mukai^1, 2^, Shin-ichi Sawada^1, 2^, Takeshi Azuma^4^, Masanori Hatakeyama^5*^, and Kazunari Akiyoshi^1, 2*^

^1^Department of Polymer Chemistry, Graduate School of Engineering, Kyoto University, 　 Katsura, Nishikyo-ku, Kyoto 615-8510, Japan

^2^JST-ERATO Akiyoshi Bio-nanotransporter Project

^3^Division of Biosciences, Functional Proteomics Center, Graduate School of Frontier Sciences, the University of Tokyo, CREST hall 1F, Institute of Medical Science, 4-6-1 Shirokanedai, Minato-ku, Tokyo 108-8639, Japan

^4^Division of Gastroenterology, Department of Internal Medicine, Kobe University Graduate School of Medicine, 7-5-1, Kusunoki-cho, Chu-o-ku, Kobe, Hyogo 650-0017, Japan

^5^Department of Microbiology, Graduate School of Medicine, the University of Tokyo, 7-3-1 Hongo, Bunkyo-Ku, Tokyo 113-0033, Japan

**
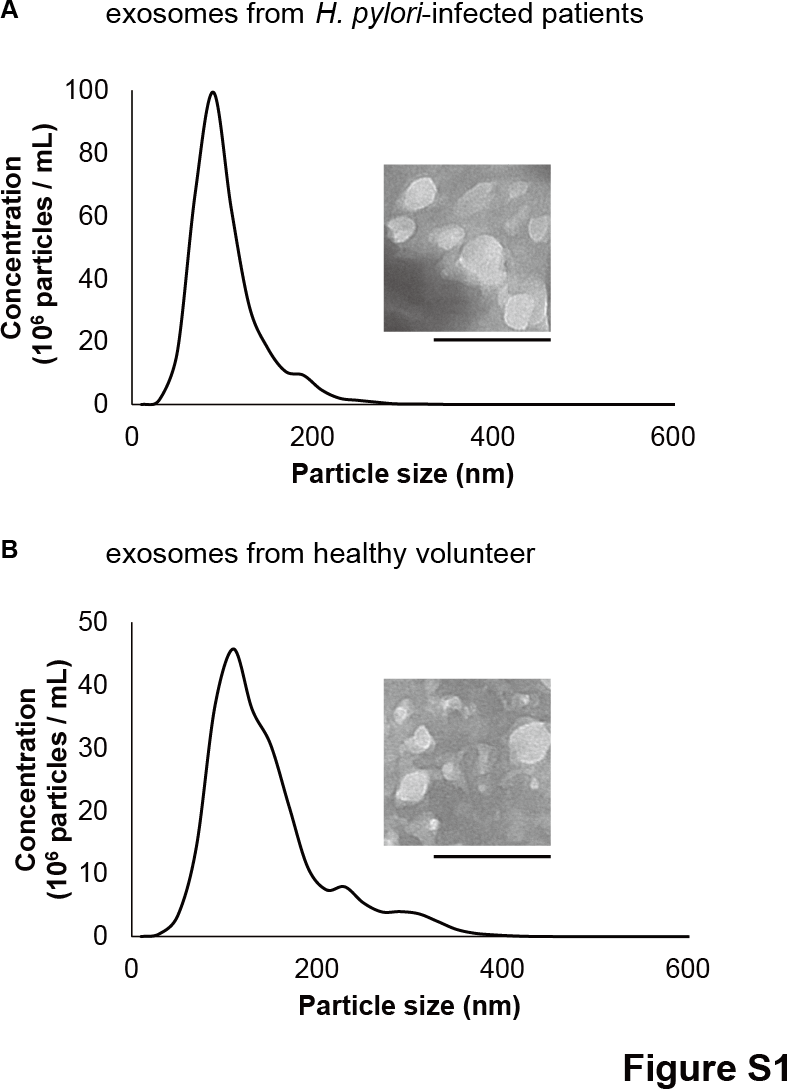
**

**Supplementary Figure S1. Size distribution and morphologies of exosome from *H. pylori*-infected patients (A) and exosome from healthy volunteers (B) were observed by Transmission electron microscopy (TEM). Scale bar = 100 nm. The average sizes of exosomes analysed by Nanoparticle Tracking analysis (NTA) were 104 nm and 144 nm, respectively.**

**Supplementary Figure S2. Mass spectrometric identification of exosomal CD63 protein in *H. pylori*-infected and *H. pylori* –uninfected human serum.**

**Exosomes were isolated from *H. pylori*-infected (A) or uninfected (B) human serum and analysed by LC-MS/MS. Extracted ion chromatograms (XICs) of CD63-derived peptides are shown. The targeted mass-to-charge ratios (m/z values) were 629.2876 (a) and 519.2396 (b), respectively. Peak IDs in a–b correspond to those in Supplementary Table S1.**

**
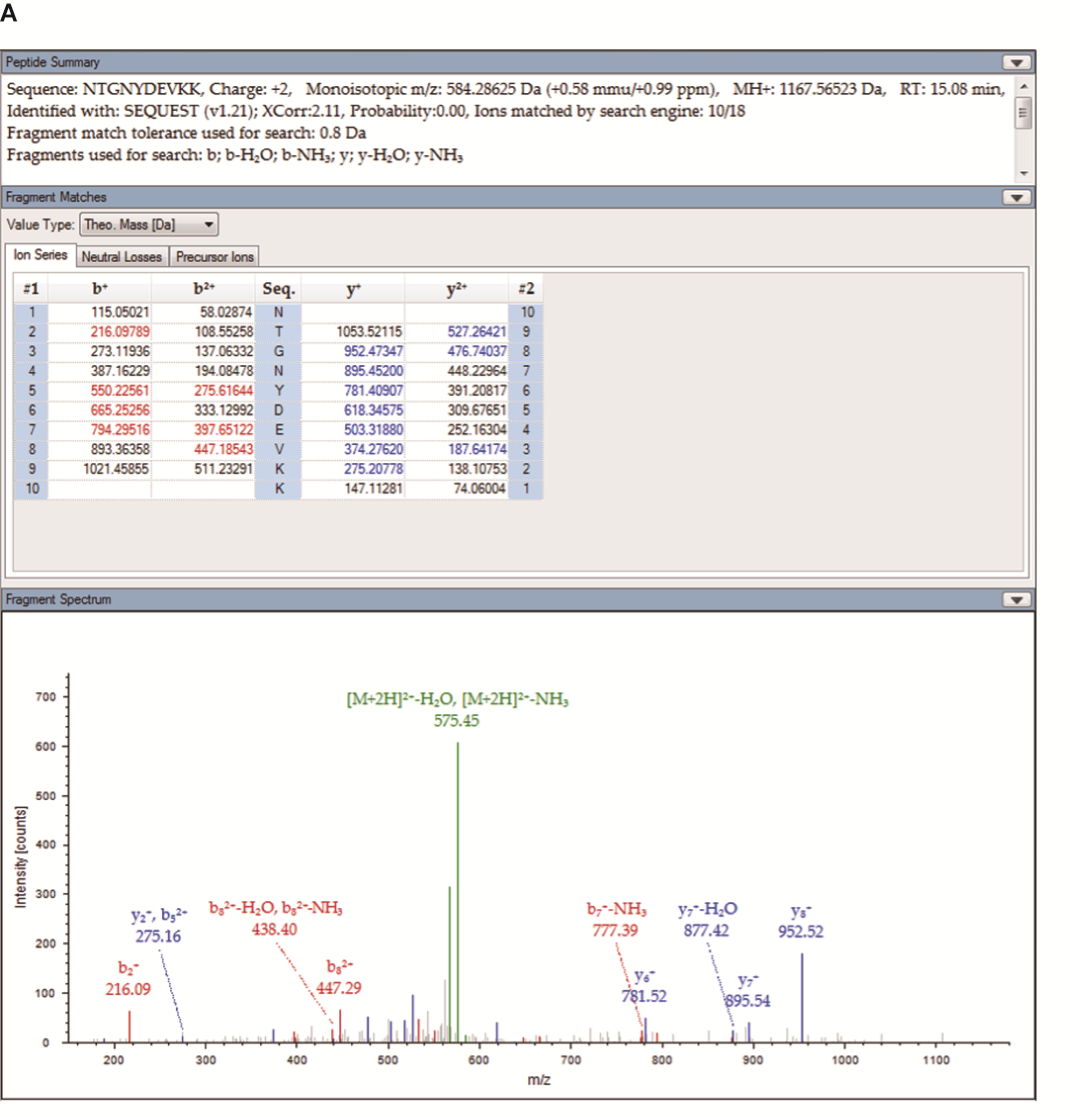
**

**Supplementary Figure S3. CagA protein identified in H. pylori - infected human serum exosomes by LC-MS/MS. (A) Mass spectrum from a tryptic peptide NTGNYDEVKK.**

**
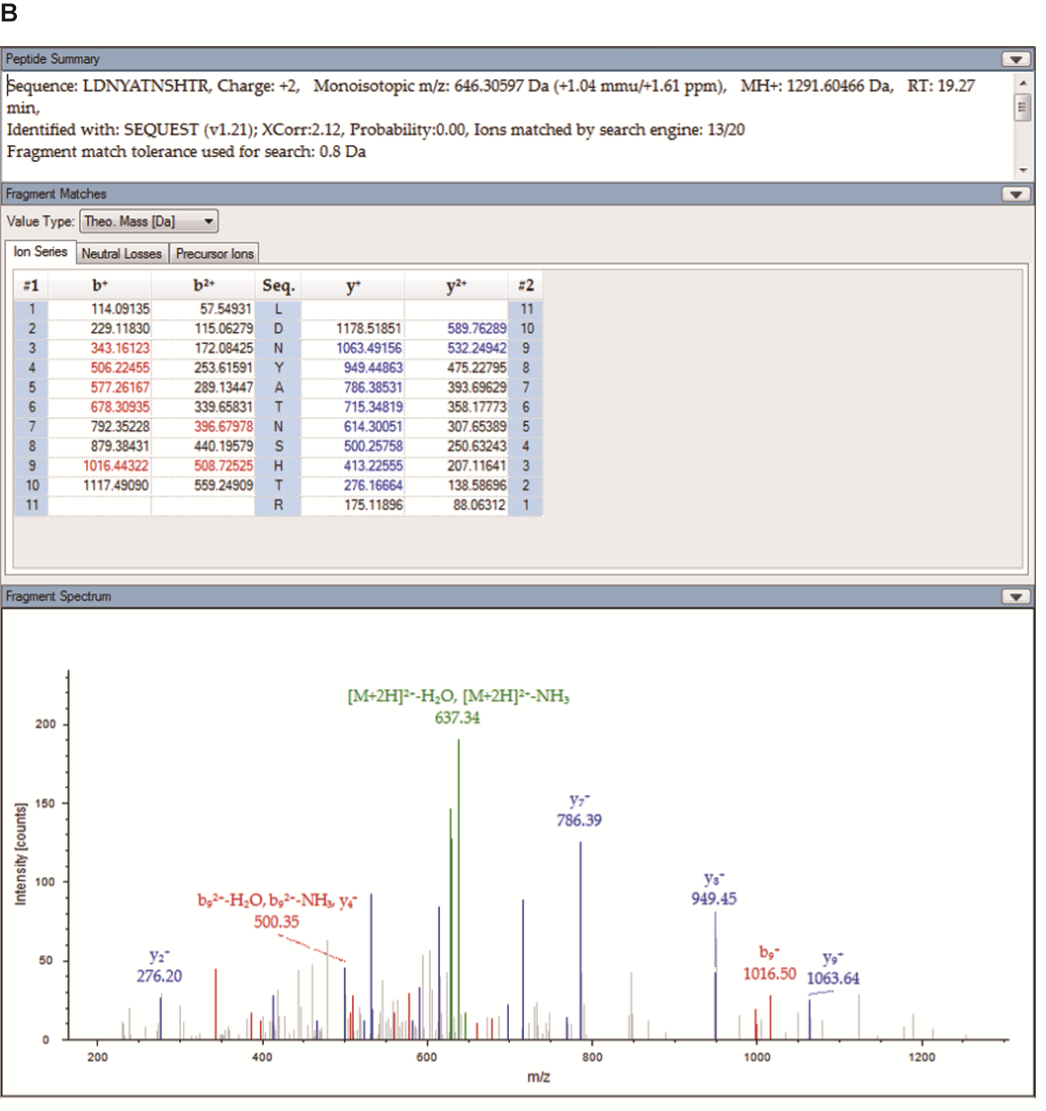
**

**Supplementary Figure S3. (B) Mass spectrum from a tryptic peptide LDNYATNSHTR.**


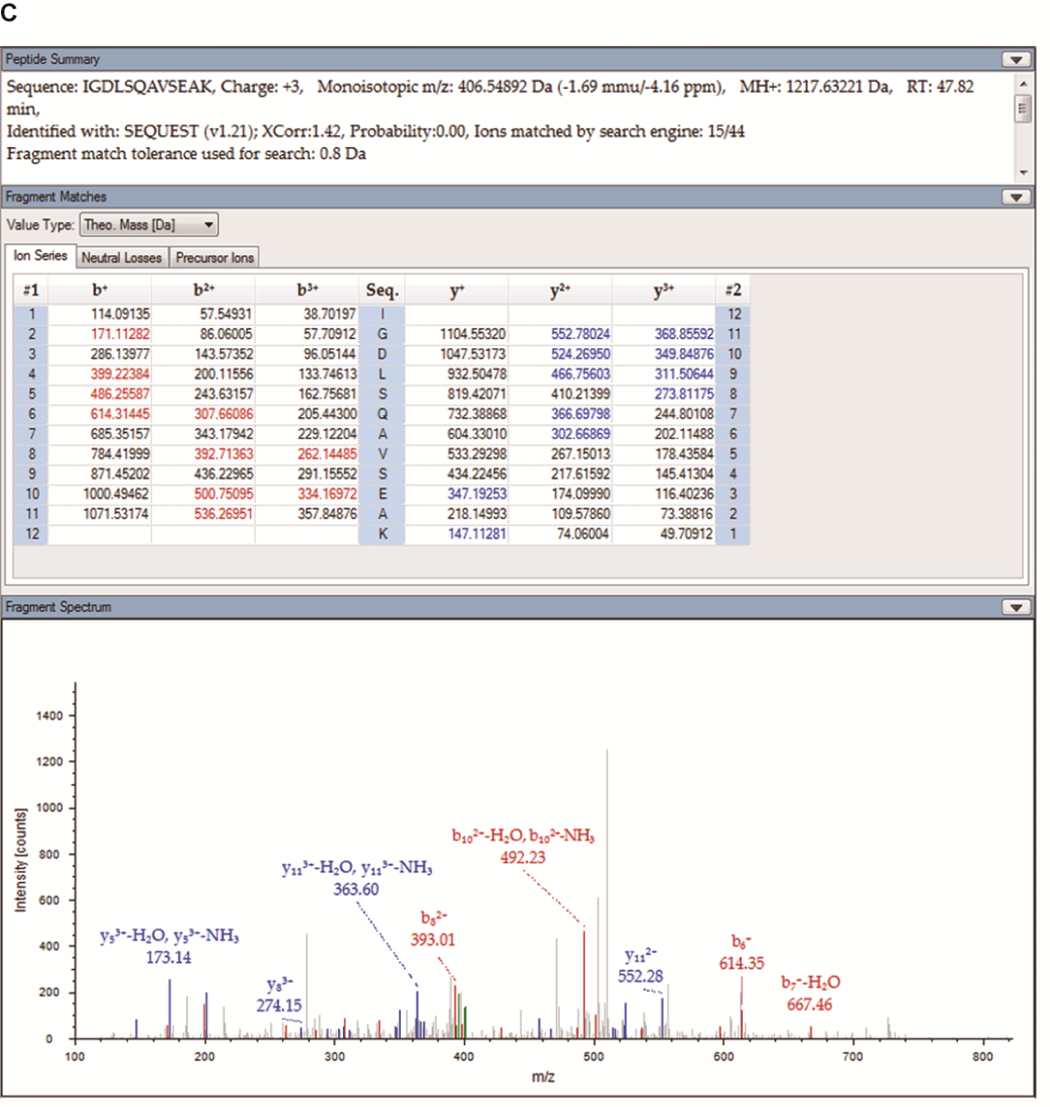
**Supplementary Figure S3. (C) Mass spectrum from a tryptic peptide** **IGDLSQAVSEAK.**

**
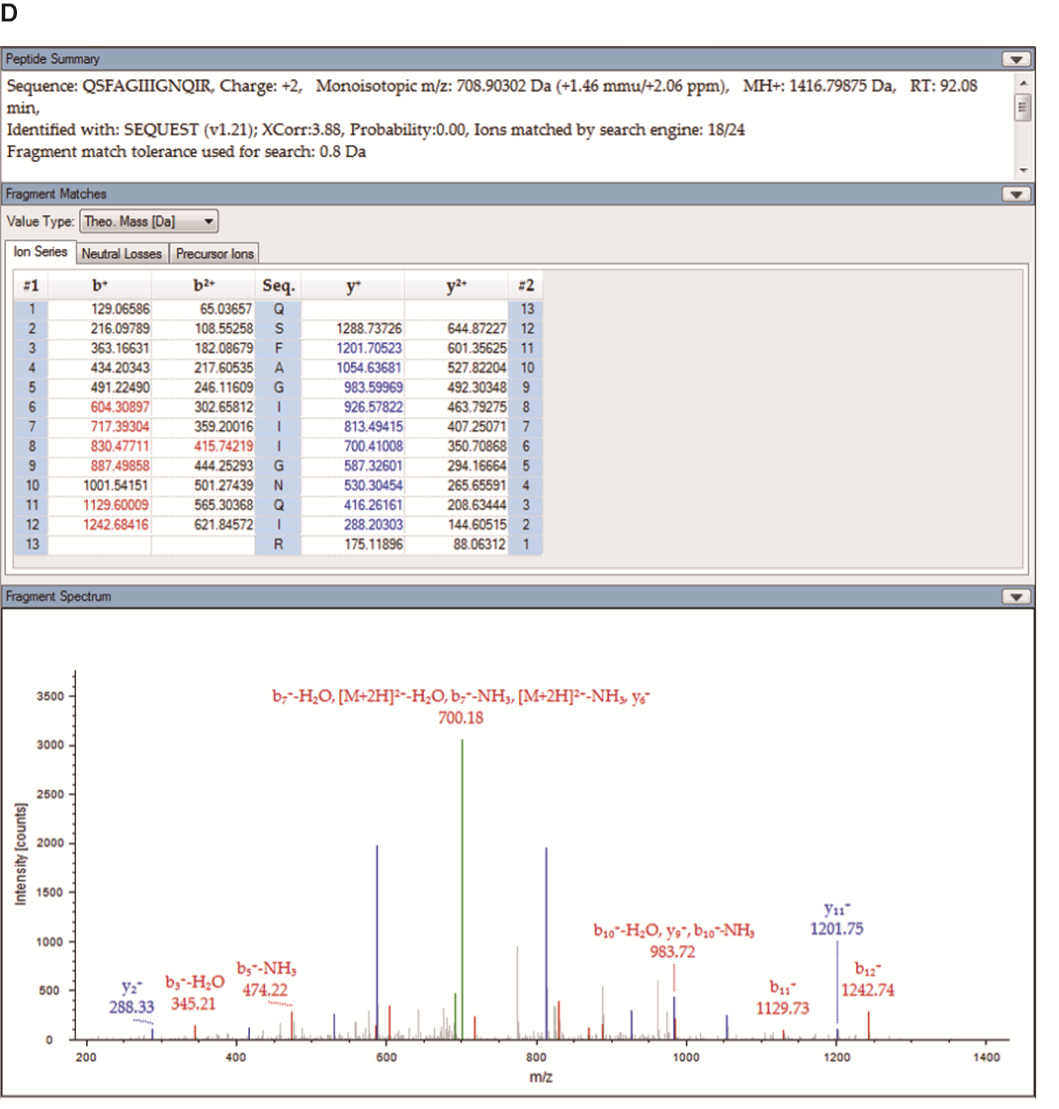
**

**Supplementary Figure S3. (D) Mass spectrum from a tryptic peptide** **QSFAGIIIGNQIR.**

**Supplementary Figure S4. Full-length western blot for Figure 2D.**

**Supplementary Figure S5. Full-length western blot for Figure 3A.**

Supplementary Table S1 CD63 protein identified in *H. pylori*-infected and *H. pylori*-uninfected human serum exosomes by LC-MS/MS.

| Peak ID^1^ | m/z^2^  [Da] | Charge | RT^3^  [min] | UniProt Entry | Protein Descriptions | amino acid number | Sequence |
| --- | --- | --- | --- | --- | --- | --- | --- |
| a | 629.2876 | 2 |  | P08962 | CD63 antigen | 111-120 | VMSEFNNNFR |
| b | 519.2396 | 2 |  | P08962 | CD63 antigen | 121-128 | QQMENYPK |
|  |  |  |  |  |  |  |  |
| 1 IDs are correspond to peaks in Supplementary Figure S2. | | | | | |  |  |
| 2 Mass-to-charge ratio. | | |  |  |  |  |  |
| 3 Retention time of nano-HPLC. | | | |  |  |  |  |

| Supplementary Table S2. Proteins in exosomes from *H. pylori*-uninfected human serum were analysed by LC-MS/MS | | | | |
| --- | --- | --- | --- | --- |
| Accession | Description | Score | Coverage | # Unique Peptides |
| P08195 | 4F2 cell-surface antigen heavy chain OS=Homo sapiens GN=SLC3A2 PE=1 SV=3 - [4F2_HUMAN] | 19.26 | 14.76% | 7 |
| P01009 | Alpha-1-antitrypsin OS=Homo sapiens GN=SERPINA1 PE=1 SV=3 - [A1AT_HUMAN] | 21.76 | 27.03% | 11 |
| P08697 | Alpha-2-antiplasmin OS=Homo sapiens GN=SERPINF2 PE=1 SV=3 - [A2AP_HUMAN] | 15.02 | 14.26% | 6 |
| P01023 | Alpha-2-macroglobulin OS=Homo sapiens GN=A2M PE=1 SV=3 - [A2MG_HUMAN] | 282.44 | 40.71% | 57 |
| P60709 | Actin, cytoplasmic 1 OS=Homo sapiens GN=ACTB PE=1 SV=1 - [ACTB_HUMAN] | 8.69 | 9.87% | 3 |
| P12814 | Alpha-actinin-1 OS=Homo sapiens GN=ACTN1 PE=1 SV=2 - [ACTN1_HUMAN] | 3.26 | 1.23% | 1 |
| P02768 | Serum albumin OS=Homo sapiens GN=ALB PE=1 SV=2 - [ALBU_HUMAN] | 181.4 | 72.91% | 49 |
| P35858 | Insulin-like growth factor-binding protein complex acid labile subunit OS=Homo sapiens GN=IGFALS PE=1 SV=1 - [ALS_HUMAN] | 5.98 | 4.79% | 2 |
| P02760 | Protein AMBP OS=Homo sapiens GN=AMBP PE=1 SV=1 - [AMBP_HUMAN] | 11.95 | 16.76% | 5 |
| P15144 | Aminopeptidase N OS=Homo sapiens GN=ANPEP PE=1 SV=4 - [AMPN_HUMAN] | 137.73 | 30.71% | 37 |
| P01019 | Angiotensinogen OS=Homo sapiens GN=AGT PE=1 SV=1 - [ANGT_HUMAN] | 49.04 | 30.52% | 14 |
| Q4KMQ2 | Anoctamin-6 OS=Homo sapiens GN=ANO6 PE=1 SV=2 - [ANO6_HUMAN] | 2.32 | 1.32% | 1 |
| P01008 | Antithrombin-III OS=Homo sapiens GN=SERPINC1 PE=1 SV=1 - [ANT3_HUMAN] | 60.14 | 45.91% | 20 |
| Q06481 | Amyloid-like protein 2 OS=Homo sapiens GN=APLP2 PE=1 SV=2 - [APLP2_HUMAN] | 2.09 | 1.18% | 1 |
| P02647 | Apolipoprotein A-I OS=Homo sapiens GN=APOA1 PE=1 SV=1 - [APOA1_HUMAN] | 7 | 8.99% | 2 |
| P04114 | Apolipoprotein B-100 OS=Homo sapiens GN=APOB PE=1 SV=2 - [APOB_HUMAN] | 54.89 | 6.62% | 25 |
| P02649 | Apolipoprotein E OS=Homo sapiens GN=APOE PE=1 SV=1 - [APOE_HUMAN] | 61.97 | 60.57% | 19 |
| O14791 | Apolipoprotein L1 OS=Homo sapiens GN=APOL1 PE=1 SV=5 - [APOL1_HUMAN] | 56 | 27.89% | 14 |
| Q13625 | Apoptosis-stimulating of p53 protein 2 OS=Homo sapiens GN=TP53BP2 PE=1 SV=2 - [ASPP2_HUMAN] | 0 | 1.68% | 1 |
| P05023 | Sodium/potassium-transporting ATPase subunit alpha-1 OS=Homo sapiens GN=ATP1A1 PE=1 SV=1 - [AT1A1_HUMAN] | 6.24 | 3.03% | 2 |
| P02730 | Band 3 anion transport protein OS=Homo sapiens GN=SLC4A1 PE=1 SV=3 - [B3AT_HUMAN] | 11.86 | 5.49% | 4 |
| Q15582 | Transforming growth factor-beta-induced protein ig-h3 OS=Homo sapiens GN=TGFBI PE=1 SV=1 - [BGH3_HUMAN] | 7.05 | 6.30% | 3 |
| P02745 | Complement C1q subcomponent subunit A OS=Homo sapiens GN=C1QA PE=1 SV=2 - [C1QA_HUMAN] | 4.81 | 18.37% | 4 |
| P02746 | Complement C1q subcomponent subunit B OS=Homo sapiens GN=C1QB PE=1 SV=3 - [C1QB_HUMAN] | 62.58 | 31.23% | 9 |
| P02747 | Complement C1q subcomponent subunit C OS=Homo sapiens GN=C1QC PE=1 SV=3 - [C1QC_HUMAN] | 47.63 | 30.20% | 10 |
| Q9BXJ4 | Complement C1q tumor necrosis factor-related protein 3 OS=Homo sapiens GN=C1QTNF3 PE=1 SV=1 - [C1QT3_HUMAN] | 15.6 | 23.17% | 7 |
| P00736 | Complement C1r subcomponent OS=Homo sapiens GN=C1R PE=1 SV=2 - [C1R_HUMAN] | 18.73 | 15.32% | 9 |
| P09871 | Complement C1s subcomponent OS=Homo sapiens GN=C1S PE=1 SV=1 - [C1S_HUMAN] | 58.64 | 27.91% | 14 |
| P04003 | C4b-binding protein alpha chain OS=Homo sapiens GN=C4BPA PE=1 SV=2 - [C4BPA_HUMAN] | 2.15 | 2.51% | 1 |
| Q01518 | Adenylyl cyclase-associated protein 1 OS=Homo sapiens GN=CAP1 PE=1 SV=5 - [CAP1_HUMAN] | 6.69 | 6.11% | 2 |
| P04040 | Catalase OS=Homo sapiens GN=CAT PE=1 SV=3 - [CATA_HUMAN] | 2.47 | 5.31% | 1 |
| Q8WXQ8 | Carboxypeptidase A5 OS=Homo sapiens GN=CPA5 PE=2 SV=1 - [CBPA5_HUMAN] | 0 | 4.13% | 1 |
| P16671 | Platelet glycoprotein 4 OS=Homo sapiens GN=CD36 PE=1 SV=2 - [CD36_HUMAN] | 6.17 | 3.81% | 1 |
| O43866 | CD5 antigen-like OS=Homo sapiens GN=CD5L PE=1 SV=1 - [CD5L_HUMAN] | 27.69 | 35.16% | 11 |
| P08962 | CD63 antigen OS=Homo sapiens GN=CD63 PE=1 SV=2 - [CD63_HUMAN] | 1.91 | 4.20% | 1 |
| P21926 | CD9 antigen OS=Homo sapiens GN=CD9 PE=1 SV=4 - [CD9_HUMAN] | 0 | 4.82% | 1 |
| Q9NYQ7 | Cadherin EGF LAG seven-pass G-type receptor 3 OS=Homo sapiens GN=CELSR3 PE=1 SV=2 - [CELR3_HUMAN] | 0 | 0.27% | 1 |
| P00450 | Ceruloplasmin OS=Homo sapiens GN=CP PE=1 SV=1 - [CERU_HUMAN] | 211.97 | 41.13% | 47 |
| P00751 | Complement factor B OS=Homo sapiens GN=CFB PE=1 SV=2 - [CFAB_HUMAN] | 2.92 | 1.83% | 1 |
| P08603 | Complement factor H OS=Homo sapiens GN=CFH PE=1 SV=4 - [CFAH_HUMAN] | 157.61 | 43.87% | 48 |
| P06276 | Cholinesterase OS=Homo sapiens GN=BCHE PE=1 SV=1 - [CHLE_HUMAN] | 12.54 | 9.14% | 5 |
| P10909 | Clusterin OS=Homo sapiens GN=CLU PE=1 SV=1 - [CLUS_HUMAN] | 38.41 | 34.08% | 15 |
| P01024 | Complement C3 OS=Homo sapiens GN=C3 PE=1 SV=2 - [CO3_HUMAN] | 406.35 | 52.62% | 88 |
| P0C0L4 | Complement C4-A OS=Homo sapiens GN=C4A PE=1 SV=2 - [CO4A_HUMAN] | 391.72 | 50.00% | 4 |
| P0C0L5 | Complement C4-B OS=Homo sapiens GN=C4B PE=1 SV=2 - [CO4B_HUMAN] | 390.22 | 48.34% | 2 |
| P01031 | Complement C5 OS=Homo sapiens GN=C5 PE=1 SV=4 - [CO5_HUMAN] | 19.55 | 4.77% | 7 |
| P13671 | Complement component C6 OS=Homo sapiens GN=C6 PE=1 SV=3 - [CO6_HUMAN] | 66.25 | 26.66% | 25 |
| P10643 | Complement component C7 OS=Homo sapiens GN=C7 PE=1 SV=2 - [CO7_HUMAN] | 32.46 | 16.49% | 10 |
| P07357 | Complement component C8 alpha chain OS=Homo sapiens GN=C8A PE=1 SV=2 - [CO8A_HUMAN] | 46.31 | 24.83% | 14 |
| P07358 | Complement component C8 beta chain OS=Homo sapiens GN=C8B PE=1 SV=3 - [CO8B_HUMAN] | 5.52 | 5.25% | 2 |
| P07360 | Complement component C8 gamma chain OS=Homo sapiens GN=C8G PE=1 SV=3 - [CO8G_HUMAN] | 10.47 | 42.08% | 6 |
| P02748 | Complement component C9 OS=Homo sapiens GN=C9 PE=1 SV=2 - [CO9_HUMAN] | 48.65 | 33.09% | 18 |
| Q9BWP8 | Collectin-11 OS=Homo sapiens GN=COLEC11 PE=1 SV=1 - [COL11_HUMAN] | 2.37 | 4.06% | 1 |
| Q14129 | Protein DGCR6 OS=Homo sapiens GN=DGCR6 PE=1 SV=3 - [DGCR6_HUMAN] | 0 | 7.73% | 1 |
| Q08211 | ATP-dependent RNA helicase A OS=Homo sapiens GN=DHX9 PE=1 SV=4 - [DHX9_HUMAN] | 14.93 | 3.78% | 6 |
| P09172 | Dopamine beta-hydroxylase OS=Homo sapiens GN=DBH PE=1 SV=3 - [DOPO_HUMAN] | 9.37 | 6.48% | 3 |
| P27487 | Dipeptidyl peptidase 4 OS=Homo sapiens GN=DPP4 PE=1 SV=2 - [DPP4_HUMAN] | 30.49 | 19.97% | 14 |
| P68104 | Elongation factor 1-alpha 1 OS=Homo sapiens GN=EEF1A1 PE=1 SV=1 - [EF1A1_HUMAN] | 3.5 | 4.98% | 2 |
| P26641 | Elongation factor 1-gamma OS=Homo sapiens GN=EEF1G PE=1 SV=3 - [EF1G_HUMAN] | 2.18 | 2.29% | 1 |
| P06733 | Alpha-enolase OS=Homo sapiens GN=ENO1 PE=1 SV=2 - [ENOA_HUMAN] | 2.57 | 2.76% | 1 |
| O95208 | Epsin-2 OS=Homo sapiens GN=EPN2 PE=1 SV=3 - [EPN2_HUMAN] | 0 | 2.81% | 1 |
| Q9BSJ8 | Extended synaptotagmin-1 OS=Homo sapiens GN=ESYT1 PE=1 SV=1 - [ESYT1_HUMAN] | 1.94 | 1.18% | 1 |
| Q92817 | Envoplakin OS=Homo sapiens GN=EVPL PE=1 SV=3 - [EVPL_HUMAN] | 2 | 0.44% | 1 |
| P03951 | Coagulation factor XI OS=Homo sapiens GN=F11 PE=1 SV=1 - [FA11_HUMAN] | 12.29 | 11.04% | 6 |
| Q9Y6R7 | IgGFc-binding protein OS=Homo sapiens GN=FCGBP PE=1 SV=3 - [FCGBP_HUMAN] | 72.96 | 6.11% | 24 |
| O75636 | Ficolin-3 OS=Homo sapiens GN=FCN3 PE=1 SV=2 - [FCN3_HUMAN] | 74.22 | 43.81% | 14 |
| P02671 | Fibrinogen alpha chain OS=Homo sapiens GN=FGA PE=1 SV=2 - [FIBA_HUMAN] | 21.16 | 8.66% | 6 |
| P02751 | Fibronectin OS=Homo sapiens GN=FN1 PE=1 SV=4 - [FINC_HUMAN] | 174.43 | 27.20% | 48 |
| P21333 | Filamin-A OS=Homo sapiens GN=FLNA PE=1 SV=4 - [FLNA_HUMAN] | 0 | 0.60% | 1 |
| Q04609 | Glutamate carboxypeptidase 2 OS=Homo sapiens GN=FOLH1 PE=1 SV=1 - [FOLH1_HUMAN] | 7.07 | 7.47% | 4 |
| Q9P2B2 | Prostaglandin F2 receptor negative regulator OS=Homo sapiens GN=PTGFRN PE=1 SV=2 - [FPRP_HUMAN] | 4.09 | 2.39% | 2 |
| P02794 | Ferritin heavy chain OS=Homo sapiens GN=FTH1 PE=1 SV=2 - [FRIH_HUMAN] | 11.58 | 14.75% | 3 |
| P02792 | Ferritin light chain OS=Homo sapiens GN=FTL PE=1 SV=2 - [FRIL_HUMAN] | 4.19 | 8.57% | 1 |
| P04406 | Glyceraldehyde-3-phosphate dehydrogenase OS=Homo sapiens GN=GAPDH PE=1 SV=3 - [G3P_HUMAN] | 1.91 | 7.76% | 2 |
| Q14697 | Neutral alpha-glucosidase AB OS=Homo sapiens GN=GANAB PE=1 SV=3 - [GANAB_HUMAN] | 11.82 | 6.25% | 4 |
| P06396 | Gelsolin OS=Homo sapiens GN=GSN PE=1 SV=1 - [GELS_HUMAN] | 24.11 | 18.16% | 10 |
| P13224 | Platelet glycoprotein Ib beta chain OS=Homo sapiens GN=GP1BB PE=1 SV=1 - [GP1BB_HUMAN] | 5.24 | 10.68% | 2 |
| P11021 | 78 kDa glucose-regulated protein OS=Homo sapiens GN=HSPA5 PE=1 SV=2 - [GRP78_HUMAN] | 5.35 | 4.28% | 1 |
| Q92896 | Golgi apparatus protein 1 OS=Homo sapiens GN=GLG1 PE=1 SV=2 - [GSLG1_HUMAN] | 2.65 | 0.93% | 1 |
| P11166 | Solute carrier family 2, facilitated glucose transporter member 1 OS=Homo sapiens GN=SLC2A1 PE=1 SV=2 - [GTR1_HUMAN] | 2.91 | 4.27% | 2 |
| P11169 | Solute carrier family 2, facilitated glucose transporter member 3 OS=Homo sapiens GN=SLC2A3 PE=1 SV=1 - [GTR3_HUMAN] | 1.66 | 1.41% | 1 |
| P16403 | Histone H1.2 OS=Homo sapiens GN=HIST1H1C PE=1 SV=2 - [H12_HUMAN] | 6.08 | 9.86% | 2 |
| Q96KK5 | Histone H2A type 1-H OS=Homo sapiens GN=HIST1H2AH PE=1 SV=3 - [H2A1H_HUMAN] | 9.8 | 27.34% | 3 |
| P62805 | Histone H4 OS=Homo sapiens GN=HIST1H4A PE=1 SV=2 - [H4_HUMAN] | 6.13 | 31.07% | 3 |
| P69905 | Hemoglobin subunit alpha OS=Homo sapiens GN=HBA1 PE=1 SV=2 - [HBA_HUMAN] | 13.03 | 16.90% | 2 |
| P68871 | Hemoglobin subunit beta OS=Homo sapiens GN=HBB PE=1 SV=2 - [HBB_HUMAN] | 7.9 | 29.25% | 3 |
| P05546 | Heparin cofactor 2 OS=Homo sapiens GN=SERPIND1 PE=1 SV=3 - [HEP2_HUMAN] | 2.85 | 1.80% | 1 |
| Q04756 | Hepatocyte growth factor activator OS=Homo sapiens GN=HGFAC PE=1 SV=1 - [HGFA_HUMAN] | 32.66 | 15.88% | 9 |
| Q00839 | Heterogeneous nuclear ribonucleoprotein U OS=Homo sapiens GN=HNRNPU PE=1 SV=6 - [HNRPU_HUMAN] | 6.93 | 4.97% | 3 |
| P00739 | Haptoglobin-related protein OS=Homo sapiens GN=HPR PE=2 SV=2 - [HPTR_HUMAN] | 86.42 | 50.57% | 8 |
| P00738 | Haptoglobin OS=Homo sapiens GN=HP PE=1 SV=1 - [HPT_HUMAN] | 79.38 | 37.68% | 7 |
| P04196 | Histidine-rich glycoprotein OS=Homo sapiens GN=HRG PE=1 SV=1 - [HRG_HUMAN] | 17.75 | 9.14% | 4 |
| O95757 | Heat shock 70 kDa protein 4L OS=Homo sapiens GN=HSPA4L PE=1 SV=3 - [HS74L_HUMAN] | 2.95 | 1.67% | 1 |
| P11142 | Heat shock cognate 71 kDa protein OS=Homo sapiens GN=HSPA8 PE=1 SV=1 - [HSP7C_HUMAN] | 15.32 | 12.38% | 4 |
| P01743 | Ig heavy chain V-I region HG3 OS=Homo sapiens PE=3 SV=1 - [HV102_HUMAN] | 5.65 | 18.80% | 1 |
| P23083 | Ig heavy chain V-I region V35 OS=Homo sapiens PE=1 SV=1 - [HV103_HUMAN] | 12.65 | 40.17% | 3 |
| P01825 | Ig heavy chain V-II region NEWM OS=Homo sapiens PE=1 SV=1 - [HV207_HUMAN] | 2.94 | 13.68% | 1 |
| P04438 | Ig heavy chain V-II region SESS OS=Homo sapiens PE=2 SV=1 - [HV208_HUMAN] | 4.69 | 10.88% | 2 |
| P01763 | Ig heavy chain V-III region WEA OS=Homo sapiens PE=1 SV=1 - [HV302_HUMAN] | 11.03 | 26.32% | 1 |
| P01764 | Ig heavy chain V-III region VH26 OS=Homo sapiens PE=1 SV=1 - [HV303_HUMAN] | 16.7 | 28.21% | 4 |
| P01766 | Ig heavy chain V-III region BRO OS=Homo sapiens PE=1 SV=1 - [HV305_HUMAN] | 28.44 | 25.00% | 1 |
| P01767 | Ig heavy chain V-III region BUT OS=Homo sapiens PE=1 SV=1 - [HV306_HUMAN] | 9.79 | 26.09% | 1 |
| P01773 | Ig heavy chain V-III region BUR OS=Homo sapiens PE=1 SV=1 - [HV312_HUMAN] | 5.12 | 9.24% | 1 |
| P01775 | Ig heavy chain V-III region LAY OS=Homo sapiens PE=1 SV=1 - [HV314_HUMAN] | 3.53 | 15.97% | 1 |
| P01779 | Ig heavy chain V-III region TUR OS=Homo sapiens PE=1 SV=1 - [HV318_HUMAN] | 13.85 | 26.72% | 2 |
| P01781 | Ig heavy chain V-III region GAL OS=Homo sapiens PE=1 SV=1 - [HV320_HUMAN] | 22.06 | 48.28% | 6 |
| Q9Y4L1 | Hypoxia up-regulated protein 1 OS=Homo sapiens GN=HYOU1 PE=1 SV=1 - [HYOU1_HUMAN] | 2.44 | 2.20% | 2 |
| P05155 | Plasma protease C1 inhibitor OS=Homo sapiens GN=SERPING1 PE=1 SV=2 - [IC1_HUMAN] | 16.54 | 15.20% | 7 |
| P01876 | Ig alpha-1 chain C region OS=Homo sapiens GN=IGHA1 PE=1 SV=2 - [IGHA1_HUMAN] | 499.2 | 67.14% | 10 |
| P01877 | Ig alpha-2 chain C region OS=Homo sapiens GN=IGHA2 PE=1 SV=3 - [IGHA2_HUMAN] | 315.29 | 56.47% | 5 |
| P01857 | Ig gamma-1 chain C region OS=Homo sapiens GN=IGHG1 PE=1 SV=1 - [IGHG1_HUMAN] | 110.5 | 67.58% | 9 |
| P01859 | Ig gamma-2 chain C region OS=Homo sapiens GN=IGHG2 PE=1 SV=2 - [IGHG2_HUMAN] | 106.53 | 53.99% | 11 |
| P01860 | Ig gamma-3 chain C region OS=Homo sapiens GN=IGHG3 PE=1 SV=2 - [IGHG3_HUMAN] | 92.48 | 49.87% | 7 |
| P01861 | Ig gamma-4 chain C region OS=Homo sapiens GN=IGHG4 PE=1 SV=1 - [IGHG4_HUMAN] | 57.64 | 39.14% | 2 |
| P01871 | Ig mu chain C region OS=Homo sapiens GN=IGHM PE=1 SV=3 - [IGHM_HUMAN] | 59.07 | 35.40% | 14 |
| P01591 | Immunoglobulin J chain OS=Homo sapiens GN=IGJ PE=1 SV=4 - [IGJ_HUMAN] | 33.42 | 44.03% | 9 |
| P01834 | Ig kappa chain C region OS=Homo sapiens GN=IGKC PE=1 SV=1 - [IGKC_HUMAN] | 103.84 | 100.00% | 10 |
| B9A064 | Immunoglobulin lambda-like polypeptide 5 OS=Homo sapiens GN=IGLL5 PE=2 SV=2 - [IGLL5_HUMAN] | 87.04 | 42.06% | 6 |
| P05154 | Plasma serine protease inhibitor OS=Homo sapiens GN=SERPINA5 PE=1 SV=3 - [IPSP_HUMAN] | 72.09 | 39.90% | 18 |
| P08514 | Integrin alpha-IIb OS=Homo sapiens GN=ITGA2B PE=1 SV=3 - [ITA2B_HUMAN] | 136.8 | 33.49% | 31 |
| P17301 | Integrin alpha-2 OS=Homo sapiens GN=ITGA2 PE=1 SV=1 - [ITA2_HUMAN] | 0 | 1.52% | 1 |
| P26006 | Integrin alpha-3 OS=Homo sapiens GN=ITGA3 PE=1 SV=5 - [ITA3_HUMAN] | 4.94 | 1.81% | 2 |
| P08648 | Integrin alpha-5 OS=Homo sapiens GN=ITGA5 PE=1 SV=2 - [ITA5_HUMAN] | 1.95 | 1.81% | 2 |
| P11215 | Integrin alpha-M OS=Homo sapiens GN=ITGAM PE=1 SV=2 - [ITAM_HUMAN] | 2.56 | 0.95% | 1 |
| P05556 | Integrin beta-1 OS=Homo sapiens GN=ITGB1 PE=1 SV=2 - [ITB1_HUMAN] | 38.31 | 17.42% | 13 |
| P05107 | Integrin beta-2 OS=Homo sapiens GN=ITGB2 PE=1 SV=2 - [ITB2_HUMAN] | 14.2 | 9.62% | 6 |
| P05106 | Integrin beta-3 OS=Homo sapiens GN=ITGB3 PE=1 SV=2 - [ITB3_HUMAN] | 7.88 | 4.57% | 2 |
| P19827 | Inter-alpha-trypsin inhibitor heavy chain H1 OS=Homo sapiens GN=ITIH1 PE=1 SV=3 - [ITIH1_HUMAN] | 11.99 | 4.83% | 4 |
| P19823 | Inter-alpha-trypsin inhibitor heavy chain H2 OS=Homo sapiens GN=ITIH2 PE=1 SV=2 - [ITIH2_HUMAN] | 35.71 | 14.48% | 13 |
| Q06033 | Inter-alpha-trypsin inhibitor heavy chain H3 OS=Homo sapiens GN=ITIH3 PE=1 SV=2 - [ITIH3_HUMAN] | 7.88 | 5.62% | 4 |
| Q14624 | Inter-alpha-trypsin inhibitor heavy chain H4 OS=Homo sapiens GN=ITIH4 PE=1 SV=4 - [ITIH4_HUMAN] | 121.03 | 30.54% | 36 |
| O43736 | Integral membrane protein 2A OS=Homo sapiens GN=ITM2A PE=1 SV=2 - [ITM2A_HUMAN] | 0 | 11.03% | 1 |
| Q8IV33 | Uncharacterized protein KIAA0825 OS=Homo sapiens GN=KIAA0825 PE=2 SV=2 - [K0825_HUMAN] | 2.05 | 0.63% | 1 |
| Q8WUJ3 | Cell migration-inducing and hyaluronan-binding protein OS=Homo sapiens GN=CEMIP PE=1 SV=2 - [CEMIP_HUMAN] | 32.97 | 10.80% | 13 |
| Q8N163 | Cell cycle and apoptosis regulator protein 2 OS=Homo sapiens GN=CCAR2 PE=1 SV=2 - [CCAR2_HUMAN] | 2.82 | 6.07% | 3 |
| P07288 | Prostate-specific antigen OS=Homo sapiens GN=KLK3 PE=1 SV=2 - [KLK3_HUMAN] | 8.87 | 25.67% | 3 |
| P03952 | Plasma kallikrein OS=Homo sapiens GN=KLKB1 PE=1 SV=1 - [KLKB1_HUMAN] | 148.59 | 52.66% | 44 |
| P01042 | Kininogen-1 OS=Homo sapiens GN=KNG1 PE=1 SV=2 - [KNG1_HUMAN] | 1.66 | 2.02% | 1 |
| P14618 | Pyruvate kinase PKM OS=Homo sapiens GN=PKM PE=1 SV=4 - [KPYM_HUMAN] | 3.15 | 2.45% | 1 |
| P01597 | Ig kappa chain V-I region DEE OS=Homo sapiens PE=1 SV=1 - [KV105_HUMAN] | 10.54 | 22.22% | 2 |
| P01598 | Ig kappa chain V-I region EU OS=Homo sapiens PE=1 SV=1 - [KV106_HUMAN] | 14.36 | 32.41% | 3 |
| P01602 | Ig kappa chain V-I region HK102 (Fragment) OS=Homo sapiens GN=IGKV1-5 PE=4 SV=1 - [KV110_HUMAN] | 0 | 15.38% | 1 |
| P01605 | Ig kappa chain V-I region Lay OS=Homo sapiens PE=1 SV=1 - [KV113_HUMAN] | 5.06 | 8.33% | 1 |
| P01610 | Ig kappa chain V-I region WEA OS=Homo sapiens PE=1 SV=1 - [KV118_HUMAN] | 8.99 | 36.11% | 3 |
| P01611 | Ig kappa chain V-I region Wes OS=Homo sapiens PE=1 SV=1 - [KV119_HUMAN] | 7.53 | 16.67% | 1 |
| P01614 | Ig kappa chain V-II region Cum OS=Homo sapiens PE=1 SV=1 - [KV201_HUMAN] | 11.67 | 39.13% | 1 |
| P01615 | Ig kappa chain V-II region FR OS=Homo sapiens PE=1 SV=1 - [KV202_HUMAN] | 1.76 | 7.08% | 1 |
| P01616 | Ig kappa chain V-II region MIL OS=Homo sapiens PE=1 SV=1 - [KV203_HUMAN] | 2.58 | 21.43% | 1 |
| P01617 | Ig kappa chain V-II region TEW OS=Homo sapiens PE=1 SV=1 - [KV204_HUMAN] | 15.79 | 38.94% | 1 |
| P06310 | Ig kappa chain V-II region RPMI 6410 OS=Homo sapiens PE=4 SV=1 - [KV206_HUMAN] | 11.61 | 18.05% | 3 |
| P01620 | Ig kappa chain V-III region SIE OS=Homo sapiens PE=1 SV=1 - [KV302_HUMAN] | 36.32 | 50.46% | 3 |
| P04433 | Ig kappa chain V-III region VG (Fragment) OS=Homo sapiens PE=1 SV=1 - [KV309_HUMAN] | 8.36 | 32.17% | 3 |
| P18135 | Ig kappa chain V-III region HAH OS=Homo sapiens PE=2 SV=1 - [KV312_HUMAN] | 9.81 | 33.33% | 2 |
| P06312 | Ig kappa chain V-IV region (Fragment) OS=Homo sapiens GN=IGKV4-1 PE=4 SV=1 - [KV401_HUMAN] | 5.94 | 17.36% | 1 |
| P01625 | Ig kappa chain V-IV region Len OS=Homo sapiens PE=1 SV=2 - [KV402_HUMAN] | 14.95 | 23.68% | 1 |
| P0CG05 | Ig lambda-2 chain C regions OS=Homo sapiens GN=IGLC2 PE=1 SV=1 - [LAC2_HUMAN] | 108.69 | 77.36% | 2 |
| P0CG06 | Ig lambda-3 chain C regions OS=Homo sapiens GN=IGLC3 PE=1 SV=1 - [LAC3_HUMAN] | 100.53 | 70.75% | 2 |
| A0M8Q6 | Ig lambda-7 chain C region OS=Homo sapiens GN=IGLC7 PE=1 SV=2 - [LAC7_HUMAN] | 55.3 | 58.49% | 2 |
| Q16363 | Laminin subunit alpha-4 OS=Homo sapiens GN=LAMA4 PE=1 SV=4 - [LAMA4_HUMAN] | 2.64 | 1.59% | 1 |
| Q01650 | Large neutral amino acids transporter small subunit 1 OS=Homo sapiens GN=SLC7A5 PE=1 SV=2 - [LAT1_HUMAN] | 2.66 | 3.55% | 1 |
| O75387 | Large neutral amino acids transporter small subunit 3 OS=Homo sapiens GN=SLC43A1 PE=1 SV=1 - [LAT3_HUMAN] | 2.04 | 3.22% | 1 |
| P18428 | Lipopolysaccharide-binding protein OS=Homo sapiens GN=LBP PE=1 SV=3 - [LBP_HUMAN] | 9.32 | 7.28% | 4 |
| P00338 | L-lactate dehydrogenase A chain OS=Homo sapiens GN=LDHA PE=1 SV=2 - [LDHA_HUMAN] | 3.93 | 3.61% | 1 |
| Q08380 | Galectin-3-binding protein OS=Homo sapiens GN=LGALS3BP PE=1 SV=1 - [LG3BP_HUMAN] | 153.07 | 46.67% | 29 |
| P42704 | Leucine-rich PPR motif-containing protein, mitochondrial OS=Homo sapiens GN=LRPPRC PE=1 SV=3 - [LPPRC_HUMAN] | 0 | 1.94% | 2 |
| Q07954 | Prolow-density lipoprotein receptor-related protein 1 OS=Homo sapiens GN=LRP1 PE=1 SV=2 - [LRP1_HUMAN] | 150.02 | 12.85% | 46 |
| P04211 | Ig lambda chain V region 4A OS=Homo sapiens PE=4 SV=1 - [LV001_HUMAN] | 2.35 | 7.69% | 1 |
| P01699 | Ig lambda chain V-I region VOR OS=Homo sapiens PE=1 SV=1 - [LV101_HUMAN] | 2.49 | 8.11% | 1 |
| P01700 | Ig lambda chain V-I region HA OS=Homo sapiens PE=1 SV=1 - [LV102_HUMAN] | 3.11 | 23.21% | 1 |
| P01702 | Ig lambda chain V-I region NIG-64 OS=Homo sapiens PE=1 SV=1 - [LV104_HUMAN] | 3.74 | 18.92% | 1 |
| P04208 | Ig lambda chain V-I region WAH OS=Homo sapiens PE=1 SV=1 - [LV106_HUMAN] | 5.38 | 26.61% | 1 |
| P01705 | Ig lambda chain V-II region NEI OS=Homo sapiens PE=1 SV=1 - [LV202_HUMAN] | 2.82 | 7.21% | 1 |
| P04209 | Ig lambda chain V-II region NIG-84 OS=Homo sapiens PE=1 SV=1 - [LV211_HUMAN] | 2.29 | 14.29% | 1 |
| P01714 | Ig lambda chain V-III region SH OS=Homo sapiens PE=1 SV=1 - [LV301_HUMAN] | 9.11 | 25.00% | 2 |
| P80748 | Ig lambda chain V-III region LOI OS=Homo sapiens PE=1 SV=1 - [LV302_HUMAN] | 13.14 | 37.84% | 3 |
| P01717 | Ig lambda chain V-IV region Hil OS=Homo sapiens PE=1 SV=1 - [LV403_HUMAN] | 9.52 | 17.76% | 1 |
| P01719 | Ig lambda chain V-V region DEL OS=Homo sapiens PE=1 SV=1 - [LV501_HUMAN] | 4.49 | 14.81% | 1 |
| P16109 | P-selectin OS=Homo sapiens GN=SELP PE=1 SV=3 - [LYAM3_HUMAN] | 5.01 | 1.93% | 1 |
| P48740 | Mannan-binding lectin serine protease 1 OS=Homo sapiens GN=MASP1 PE=1 SV=3 - [MASP1_HUMAN] | 51.12 | 23.46% | 12 |
| O00187 | Mannan-binding lectin serine protease 2 OS=Homo sapiens GN=MASP2 PE=1 SV=4 - [MASP2_HUMAN] | 11.64 | 7.58% | 4 |
| P43243 | Matrin-3 OS=Homo sapiens GN=MATR3 PE=1 SV=2 - [MATR3_HUMAN] | 2.66 | 1.06% | 1 |
| P22897 | Macrophage mannose receptor 1 OS=Homo sapiens GN=MRC1 PE=1 SV=1 - [MRC1_HUMAN] | 0 | 1.37% | 1 |
| Q8IUG5 | Unconventional myosin-XVIIIb OS=Homo sapiens GN=MYO18B PE=1 SV=1 - [MY18B_HUMAN] | 0 | 0.62% | 1 |
| Q8N699 | Myc target protein 1 OS=Homo sapiens GN=MYCT1 PE=1 SV=1 - [MYCT1_HUMAN] | 11.01 | 10.64% | 3 |
| Q9C000 | NACHT, LRR and PYD domains-containing protein 1 OS=Homo sapiens GN=NLRP1 PE=1 SV=1 - [NALP1_HUMAN] | 2.41 | 1.63% | 1 |
| P28331 | NADH-ubiquinone oxidoreductase 75 kDa subunit, mitochondrial OS=Homo sapiens GN=NDUFS1 PE=1 SV=3 - [NDUS1_HUMAN] | 0 | 2.89% | 1 |
| P08473 | Neprilysin OS=Homo sapiens GN=MME PE=1 SV=2 - [NEP_HUMAN] | 2.01 | 0.93% | 1 |
| P14543 | Nidogen-1 OS=Homo sapiens GN=NID1 PE=1 SV=3 - [NID1_HUMAN] | 2.97 | 0.88% | 1 |
| P19338 | Nucleolin OS=Homo sapiens GN=NCL PE=1 SV=3 - [NUCL_HUMAN] | 2.78 | 1.55% | 1 |
| Q8WUM4 | Programmed cell death 6-interacting protein OS=Homo sapiens GN=PDCD6IP PE=1 SV=1 - [PDC6I_HUMAN] | 1.65 | 1.27% | 1 |
| P16284 | Platelet endothelial cell adhesion molecule OS=Homo sapiens GN=PECAM1 PE=1 SV=1 - [PECA1_HUMAN] | 2.31 | 1.49% | 1 |
| P05164 | Myeloperoxidase OS=Homo sapiens GN=MPO PE=1 SV=1 - [PERM_HUMAN] | 23.73 | 18.93% | 13 |
| Q96PD5 | N-acetylmuramoyl-L-alanine amidase OS=Homo sapiens GN=PGLYRP2 PE=1 SV=1 - [PGRP2_HUMAN] | 11.7 | 10.76% | 4 |
| Q9H4M7 | Pleckstrin homology domain-containing family A member 4 OS=Homo sapiens GN=PLEKHA4 PE=1 SV=2 - [PKHA4_HUMAN] | 0 | 5.65% | 1 |
| P62937 | Peptidyl-prolyl cis-trans isomerase A OS=Homo sapiens GN=PPIA PE=1 SV=2 - [PPIA_HUMAN] | 0 | 7.27% | 1 |
| P27918 | Properdin OS=Homo sapiens GN=CFP PE=1 SV=2 - [PROP_HUMAN] | 11.12 | 7.46% | 3 |
| P07225 | Vitamin K-dependent protein S OS=Homo sapiens GN=PROS1 PE=1 SV=1 - [PROS_HUMAN] | 57.53 | 35.21% | 22 |
| P53801 | Pituitary tumor-transforming gene 1 protein-interacting protein OS=Homo sapiens GN=PTTG1IP PE=1 SV=1 - [PTTG_HUMAN] | 2.34 | 6.67% | 1 |
| Q07020 | 60S ribosomal protein L18 OS=Homo sapiens GN=RPL18 PE=1 SV=2 - [RL18_HUMAN] | 1.74 | 6.91% | 1 |
| P47914 | 60S ribosomal protein L29 OS=Homo sapiens GN=RPL29 PE=1 SV=2 - [RL29_HUMAN] | 2.45 | 9.43% | 1 |
| Q9H9A7 | RecQ-mediated genome instability protein 1 OS=Homo sapiens GN=RMI1 PE=1 SV=3 - [RMI1_HUMAN] | 2.2 | 1.44% | 1 |
| P62249 | 40S ribosomal protein S16 OS=Homo sapiens GN=RPS16 PE=1 SV=2 - [RS16_HUMAN] | 1.93 | 6.85% | 1 |
| Q13435 | Splicing factor 3B subunit 2 OS=Homo sapiens GN=SF3B2 PE=1 SV=2 - [SF3B2_HUMAN] | 0 | 1.79% | 1 |
| P23246 | Splicing factor, proline- and glutamine-rich OS=Homo sapiens GN=SFPQ PE=1 SV=2 - [SFPQ_HUMAN] | 6.14 | 2.12% | 1 |
| Q9H254 | Spectrin beta chain, non-erythrocytic 4 OS=Homo sapiens GN=SPTBN4 PE=1 SV=2 - [SPTN4_HUMAN] | 0 | 0.66% | 1 |
| P27105 | Erythrocyte band 7 integral membrane protein OS=Homo sapiens GN=STOM PE=1 SV=3 - [STOM_HUMAN] | 8.85 | 11.46% | 3 |
| P26640 | Valine--tRNA ligase OS=Homo sapiens GN=VARS PE=1 SV=4 - [SYVC_HUMAN] | 2.66 | 1.19% | 1 |
| Q9BQE3 | Tubulin alpha-1C chain OS=Homo sapiens GN=TUBA1C PE=1 SV=1 - [TBA1C_HUMAN] | 6.03 | 7.57% | 2 |
| A6NNZ2 | Tubulin beta-8 chain-like protein LOC260334 OS=Homo sapiens PE=1 SV=1 - [TBB8L_HUMAN] | 2.24 | 2.25% | 1 |
| P55072 | Transitional endoplasmic reticulum ATPase OS=Homo sapiens GN=VCP PE=1 SV=4 - [TERA_HUMAN] | 6.82 | 4.34% | 3 |
| P02786 | Transferrin receptor protein 1 OS=Homo sapiens GN=TFRC PE=1 SV=2 - [TFR1_HUMAN] | 9.2 | 5.26% | 3 |
| P00734 | Prothrombin OS=Homo sapiens GN=F2 PE=1 SV=2 - [THRB_HUMAN] | 125.28 | 39.71% | 25 |
| Q13263 | Transcription intermediary factor 1-beta OS=Homo sapiens GN=TRIM28 PE=1 SV=5 - [TIF1B_HUMAN] | 2.06 | 1.92% | 1 |
| O94886 | CSC1-like protein 1 OS=Homo sapiens GN=TMEM63A PE=1 SV=3 - [CSCL1_HUMAN] | 2.52 | 1.12% | 1 |
| Q9H0E2 | Toll-interacting protein OS=Homo sapiens GN=TOLLIP PE=1 SV=1 - [TOLIP_HUMAN] | 2.52 | 5.11% | 1 |
| P60174 | Triosephosphate isomerase OS=Homo sapiens GN=TPI1 PE=1 SV=3 - [TPIS_HUMAN] | 2.56 | 4.20% | 1 |
| P29144 | Tripeptidyl-peptidase 2 OS=Homo sapiens GN=TPP2 PE=1 SV=4 - [TPP2_HUMAN] | 0 | 0.80% | 1 |
| Q12931 | Heat shock protein 75 kDa, mitochondrial OS=Homo sapiens GN=TRAP1 PE=1 SV=3 - [TRAP1_HUMAN] | 3.26 | 1.99% | 1 |
| P02787 | Serotransferrin OS=Homo sapiens GN=TF PE=1 SV=3 - [TRFE_HUMAN] | 10.81 | 21.20% | 10 |
| P07477 | Trypsin-1 OS=Homo sapiens GN=PRSS1 PE=1 SV=1 - [TRY1_HUMAN] | 2.58 | 3.24% | 1 |
| Q9C0H2 | Protein tweety homolog 3 OS=Homo sapiens GN=TTYH3 PE=1 SV=3 - [TTYH3_HUMAN] | 19.65 | 8.99% | 4 |
| P22314 | Ubiquitin-like modifier-activating enzyme 1 OS=Homo sapiens GN=UBA1 PE=1 SV=3 - [UBA1_HUMAN] | 6.27 | 3.40% | 2 |
| P0CG48 | Polyubiquitin-C OS=Homo sapiens GN=UBC PE=1 SV=3 - [UBC_HUMAN] | 23.65 | 40.73% | 3 |
| Q9NYU2 | UDP-glucose:glycoprotein glucosyltransferase 1 OS=Homo sapiens GN=UGGT1 PE=1 SV=3 - [UGGG1_HUMAN] | 9.36 | 3.79% | 4 |
| P04004 | Vitronectin OS=Homo sapiens GN=VTN PE=1 SV=1 - [VTNC_HUMAN] | 93.05 | 36.61% | 17 |
| Q5VUA4 | Zinc finger protein 318 OS=Homo sapiens GN=ZNF318 PE=1 SV=2 - [ZN318_HUMAN] | 0 | 1.05% | 1 |
| P23229 | Integrin alpha-6 OS=Homo sapiens GN=ITGA6 PE=1 SV=5 - [ITA6_HUMAN] | 37.25 | 10.88% | 12 |

| Supplementary Table S3. Proteins in exosomes from *H. pylori*-infected human serum were analysed by LC-MS/MS | | | | |
| --- | --- | --- | --- | --- |
| Accession | Description | Score | Coverage | # Unique Peptides |
| P61981 | 14-3-3 protein gamma OS=Homo sapiens GN=YWHAG PE=1 SV=2 - [1433G_HUMAN] | 10.82 | 9.72% | 1 |
| P27348 | 14-3-3 protein theta OS=Homo sapiens GN=YWHAQ PE=1 SV=1 - [1433T_HUMAN] | 10.84 | 9.80% | 1 |
| P63104 | 14-3-3 protein zeta/delta OS=Homo sapiens GN=YWHAZ PE=1 SV=1 - [1433Z_HUMAN] | 6.05 | 9.80% | 1 |
| P10319 | HLA class I histocompatibility antigen, B-58 alpha chain OS=Homo sapiens GN=HLA-B PE=1 SV=1 - [1B58_HUMAN] | 5.75 | 8.01% | 2 |
| P08195 | 4F2 cell-surface antigen heavy chain OS=Homo sapiens GN=SLC3A2 PE=1 SV=3 - [4F2_HUMAN] | 104.27 | 39.52% | 25 |
| P21589 | 5'-nucleotidase OS=Homo sapiens GN=NT5E PE=1 SV=1 - [5NTD_HUMAN] | 18.43 | 18.99% | 8 |
| P52209 | 6-phosphogluconate dehydrogenase, decarboxylating OS=Homo sapiens GN=PGD PE=1 SV=3 - [6PGD_HUMAN] | 3.7 | 4.35% | 1 |
| P01009 | Alpha-1-antitrypsin OS=Homo sapiens GN=SERPINA1 PE=1 SV=3 - [A1AT_HUMAN] | 13.14 | 10.05% | 4 |
| P08697 | Alpha-2-antiplasmin OS=Homo sapiens GN=SERPINF2 PE=1 SV=3 - [A2AP_HUMAN] | 6.11 | 4.89% | 2 |
| P02750 | Leucine-rich alpha-2-glycoprotein OS=Homo sapiens GN=LRG1 PE=1 SV=2 - [A2GL_HUMAN] | 1.64 | 1.73% | 1 |
| P01023 | Alpha-2-macroglobulin OS=Homo sapiens GN=A2M PE=1 SV=3 - [A2MG_HUMAN] | 126.4 | 23.27% | 32 |
| Q15758 | Neutral amino acid transporter B(0) OS=Homo sapiens GN=SLC1A5 PE=1 SV=2 - [AAAT_HUMAN] | 44.6 | 30.31% | 11 |
| P22303 | Acetylcholinesterase OS=Homo sapiens GN=ACHE PE=1 SV=1 - [ACES_HUMAN] | 3.42 | 2.12% | 1 |
| P12821 | Angiotensin-converting enzyme OS=Homo sapiens GN=ACE PE=1 SV=1 - [ACE_HUMAN] | 3.83 | 2.30% | 3 |
| Q07912 | Activated CDC42 kinase 1 OS=Homo sapiens GN=TNK2 PE=1 SV=3 - [ACK1_HUMAN] | 2.8 | 1.25% | 1 |
| P60709 | Actin, cytoplasmic 1 OS=Homo sapiens GN=ACTB PE=1 SV=1 - [ACTB_HUMAN] | 102.39 | 58.40% | 8 |
| P68032 | Actin, alpha cardiac muscle 1 OS=Homo sapiens GN=ACTC1 PE=1 SV=1 - [ACTC_HUMAN] | 61.53 | 35.28% | 2 |
| O43707 | Alpha-actinin-4 OS=Homo sapiens GN=ACTN4 PE=1 SV=2 - [ACTN4_HUMAN] | 6.64 | 3.95% | 3 |
| O14672 | Disintegrin and metalloproteinase domain-containing protein 10 OS=Homo sapiens GN=ADAM10 PE=1 SV=1 - [ADA10_HUMAN] | 29.05 | 14.30% | 8 |
| Q13443 | Disintegrin and metalloproteinase domain-containing protein 9 OS=Homo sapiens GN=ADAM9 PE=1 SV=1 - [ADAM9_HUMAN] | 2.07 | 6.47% | 3 |
| Q96JD6 | 1,5-anhydro-D-fructose reductase OS=Homo sapiens GN=AKR1E2 PE=1 SV=2 - [AKCL2_HUMAN] | 2.7 | 2.50% | 1 |
| P02768 | Serum albumin OS=Homo sapiens GN=ALB PE=1 SV=2 - [ALBU_HUMAN] | 167.69 | 65.85% | 40 |
| P35858 | Insulin-like growth factor-binding protein complex acid labile subunit OS=Homo sapiens GN=IGFALS PE=1 SV=1 - [ALS_HUMAN] | 2.62 | 2.48% | 1 |
| P02760 | Protein AMBP OS=Homo sapiens GN=AMBP PE=1 SV=1 - [AMBP_HUMAN] | 13.56 | 13.07% | 3 |
| P15144 | Aminopeptidase N OS=Homo sapiens GN=ANPEP PE=1 SV=4 - [AMPN_HUMAN] | 55.78 | 13.13% | 13 |
| P04745 | Alpha-amylase 1 OS=Homo sapiens GN=AMY1A PE=1 SV=2 - [AMY1_HUMAN] | 9.64 | 7.44% | 3 |
| P01019 | Angiotensinogen OS=Homo sapiens GN=AGT PE=1 SV=1 - [ANGT_HUMAN] | 3.24 | 7.84% | 2 |
| Q4KMQ2 | Anoctamin-6 OS=Homo sapiens GN=ANO6 PE=1 SV=2 - [ANO6_HUMAN] | 14.24 | 6.59% | 5 |
| P01008 | Antithrombin-III OS=Homo sapiens GN=SERPINC1 PE=1 SV=1 - [ANT3_HUMAN] | 79.23 | 34.48% | 17 |
| P07355 | Annexin A2 OS=Homo sapiens GN=ANXA2 PE=1 SV=2 - [ANXA2_HUMAN] | 23.72 | 28.02% | 7 |
| Q16853 | Membrane primary amine oxidase OS=Homo sapiens GN=AOC3 PE=1 SV=3 - [AOC3_HUMAN] | 2.44 | 1.05% | 1 |
| O95782 | AP-2 complex subunit alpha-1 OS=Homo sapiens GN=AP2A1 PE=1 SV=3 - [AP2A1_HUMAN] | 10.43 | 5.83% | 5 |
| P63010 | AP-2 complex subunit beta OS=Homo sapiens GN=AP2B1 PE=1 SV=1 - [AP2B1_HUMAN] | 2.32 | 1.17% | 1 |
| Q9HDC9 | Adipocyte plasma membrane-associated protein OS=Homo sapiens GN=APMAP PE=1 SV=2 - [APMAP_HUMAN] | 2.26 | 1.68% | 1 |
| P04114 | Apolipoprotein B-100 OS=Homo sapiens GN=APOB PE=1 SV=2 - [APOB_HUMAN] | 21.22 | 2.61% | 9 |
| P02649 | Apolipoprotein E OS=Homo sapiens GN=APOE PE=1 SV=1 - [APOE_HUMAN] | 16.8 | 14.51% | 4 |
| O14791 | Apolipoprotein L1 OS=Homo sapiens GN=APOL1 PE=1 SV=5 - [APOL1_HUMAN] | 19.38 | 16.83% | 5 |
| O95445 | Apolipoprotein M OS=Homo sapiens GN=APOM PE=1 SV=2 - [APOM_HUMAN] | 3.6 | 6.91% | 2 |
| P18085 | ADP-ribosylation factor 4 OS=Homo sapiens GN=ARF4 PE=1 SV=3 - [ARF4_HUMAN] | 2.2 | 11.11% | 1 |
| O95154 | Aflatoxin B1 aldehyde reductase member 3 OS=Homo sapiens GN=AKR7A3 PE=1 SV=2 - [ARK73_HUMAN] | 1.66 | 3.02% | 1 |
| Q8N5I2 | Arrestin domain-containing protein 1 OS=Homo sapiens GN=ARRDC1 PE=1 SV=1 - [ARRD1_HUMAN] | 3.33 | 3.23% | 1 |
| P07307 | Asialoglycoprotein receptor 2 OS=Homo sapiens GN=ASGR2 PE=1 SV=2 - [ASGR2_HUMAN] | 0 | 9.32% | 1 |
| P00966 | Argininosuccinate synthase OS=Homo sapiens GN=ASS1 PE=1 SV=2 - [ASSY_HUMAN] | 2.15 | 3.40% | 1 |
| Q9H7F0 | Probable cation-transporting ATPase 13A3 OS=Homo sapiens GN=ATP13A3 PE=1 SV=4 - [AT133_HUMAN] | 2.78 | 0.82% | 1 |
| P05023 | Sodium/potassium-transporting ATPase subunit alpha-1 OS=Homo sapiens GN=ATP1A1 PE=1 SV=1 - [AT1A1_HUMAN] | 111.95 | 37.15% | 31 |
| P20020 | Plasma membrane calcium-transporting ATPase 1 OS=Homo sapiens GN=ATP2B1 PE=1 SV=3 - [AT2B1_HUMAN] | 20.48 | 6.04% | 8 |
| O75882 | Attractin OS=Homo sapiens GN=ATRN PE=1 SV=2 - [ATRN_HUMAN] | 22.18 | 6.79% | 8 |
| Q76LX8 | A disintegrin and metalloproteinase with thrombospondin motifs 13 OS=Homo sapiens GN=ADAMTS13 PE=1 SV=1 - [ATS13_HUMAN] | 7.55 | 0.77% | 2 |
| P02730 | Band 3 anion transport protein OS=Homo sapiens GN=SLC4A1 PE=1 SV=3 - [B3AT_HUMAN] | 5.64 | 0.99% | 1 |
| O43505 | Beta-1,4-glucuronyltransferase 1 OS=Homo sapiens GN=B4GAT1 PE=1 SV=1 - [B4GA1_HUMAN] | 7 | 9.64% | 3 |
| Q9UQB8 | Brain-specific angiogenesis inhibitor 1-associated protein 2 OS=Homo sapiens GN=BAIAP2 PE=1 SV=1 - [BAIP2_HUMAN] | 62.2 | 50.00% | 22 |
| P35613 | Basigin OS=Homo sapiens GN=BSG PE=1 SV=2 - [BASI_HUMAN] | 13.16 | 18.44% | 5 |
| Q15582 | Transforming growth factor-beta-induced protein ig-h3 OS=Homo sapiens GN=TGFBI PE=1 SV=1 - [BGH3_HUMAN] | 6.38 | 3.81% | 2 |
| Q9UHR4 | Brain-specific angiogenesis inhibitor 1-associated protein 2-like protein 1 OS=Homo sapiens GN=BAIAP2L1 PE=1 SV=2 - [BI2L1_HUMAN] | 7.43 | 7.24% | 2 |
| Q5VW32 | BRO1 domain-containing protein BROX OS=Homo sapiens GN=BROX PE=1 SV=1 - [BROX_HUMAN] | 2.49 | 4.14% | 1 |
| Q7KYR7 | Butyrophilin subfamily 2 member A1 OS=Homo sapiens GN=BTN2A1 PE=1 SV=3 - [BT2A1_HUMAN] | 3.05 | 2.28% | 1 |
| P02745 | Complement C1q subcomponent subunit A OS=Homo sapiens GN=C1QA PE=1 SV=2 - [C1QA_HUMAN] | 2.52 | 14.29% | 3 |
| P02746 | Complement C1q subcomponent subunit B OS=Homo sapiens GN=C1QB PE=1 SV=3 - [C1QB_HUMAN] | 23.2 | 20.16% | 5 |
| P02747 | Complement C1q subcomponent subunit C OS=Homo sapiens GN=C1QC PE=1 SV=3 - [C1QC_HUMAN] | 24.08 | 30.20% | 8 |
| Q9BXJ4 | Complement C1q tumor necrosis factor-related protein 3 OS=Homo sapiens GN=C1QTNF3 PE=1 SV=1 - [C1QT3_HUMAN] | 40.17 | 23.17% | 7 |
| P00736 | Complement C1r subcomponent OS=Homo sapiens GN=C1R PE=1 SV=2 - [C1R_HUMAN] | 6.29 | 6.10% | 4 |
| P09871 | Complement C1s subcomponent OS=Homo sapiens GN=C1S PE=1 SV=1 - [C1S_HUMAN] | 17.72 | 8.43% | 4 |
| P11586 | C-1-tetrahydrofolate synthase, cytoplasmic OS=Homo sapiens GN=MTHFD1 PE=1 SV=3 - [C1TC_HUMAN] | 1.72 | 0.75% | 1 |
| Q6UB35 | Monofunctional C1-tetrahydrofolate synthase, mitochondrial OS=Homo sapiens GN=MTHFD1L PE=1 SV=1 - [C1TM_HUMAN] | 0 | 2.66% | 1 |
| Q6P1N0 | Coiled-coil and C2 domain-containing protein 1A OS=Homo sapiens GN=CC2D1A PE=1 SV=1 - [C2D1A_HUMAN] | 9.95 | 3.58% | 2 |
| P12830 | Cadherin-1 OS=Homo sapiens GN=CDH1 PE=1 SV=3 - [CADH1_HUMAN] | 17.08 | 6.80% | 4 |
| P55980 | Cytotoxicity-associated immunodominant antigen OS=Helicobacter pylori (strain ATCC 700392 / 26695) GN=cagA PE=1 SV=1 - [CAGA_HELPY] | 13.69 | 6.07% | 1 |
| P23280 | Carbonic anhydrase 6 OS=Homo sapiens GN=CA6 PE=1 SV=3 - [CAH6_HUMAN] | 1.83 | 2.92% | 1 |
| Q9Y6Q1 | Calpain-6 OS=Homo sapiens GN=CAPN6 PE=1 SV=2 - [CAN6_HUMAN] | 1.95 | 1.56% | 1 |
| Q9Y6W3 | Calpain-7 OS=Homo sapiens GN=CAPN7 PE=1 SV=1 - [CAN7_HUMAN] | 7.3 | 3.81% | 2 |
| Q01518 | Adenylyl cyclase-associated protein 1 OS=Homo sapiens GN=CAP1 PE=1 SV=5 - [CAP1_HUMAN] | 11.58 | 16.00% | 5 |
| Q6P4E1 | Protein CASC4 OS=Homo sapiens GN=CASC4 PE=1 SV=1 - [CASC4_HUMAN] | 4.54 | 2.08% | 1 |
| P15169 | Carboxypeptidase N catalytic chain OS=Homo sapiens GN=CPN1 PE=1 SV=1 - [CBPN_HUMAN] | 13.6 | 8.52% | 3 |
| Q5T0U0 | Coiled-coil domain-containing protein 122 OS=Homo sapiens GN=CCDC122 PE=1 SV=1 - [CC122_HUMAN] | 2.31 | 4.03% | 1 |
| P48509 | CD151 antigen OS=Homo sapiens GN=CD151 PE=1 SV=3 - [CD151_HUMAN] | 5.65 | 9.88% | 2 |
| Q13740 | CD166 antigen OS=Homo sapiens GN=ALCAM PE=1 SV=2 - [CD166_HUMAN] | 4.61 | 2.23% | 1 |
| Q5ZPR3 | CD276 antigen OS=Homo sapiens GN=CD276 PE=1 SV=1 - [CD276_HUMAN] | 3.41 | 2.43% | 1 |
| O43866 | CD5 antigen-like OS=Homo sapiens GN=CD5L PE=1 SV=1 - [CD5L_HUMAN] | 4.75 | 9.80% | 2 |
| P08962 | CD63 antigen OS=Homo sapiens GN=CD63 PE=1 SV=2 - [CD63_HUMAN] | 5.43 | 4.20% | 1 |
| P60033 | CD81 antigen OS=Homo sapiens GN=CD81 PE=1 SV=1 - [CD81_HUMAN] | 20.28 | 26.27% | 4 |
| P27701 | CD82 antigen OS=Homo sapiens GN=CD82 PE=1 SV=1 - [CD82_HUMAN] | 4.71 | 3.37% | 1 |
| P21926 | CD9 antigen OS=Homo sapiens GN=CD9 PE=1 SV=4 - [CD9_HUMAN] | 12.33 | 20.18% | 3 |
| P60953 | Cell division control protein 42 homolog OS=Homo sapiens GN=CDC42 PE=1 SV=2 - [CDC42_HUMAN] | 2.49 | 8.90% | 1 |
| Q9H5V8 | CUB domain-containing protein 1 OS=Homo sapiens GN=CDCP1 PE=1 SV=3 - [CDCP1_HUMAN] | 17.42 | 8.01% | 6 |
| Q53EZ4 | Centrosomal protein of 55 kDa OS=Homo sapiens GN=CEP55 PE=1 SV=3 - [CEP55_HUMAN] | 2.35 | 3.02% | 1 |
| P00450 | Ceruloplasmin OS=Homo sapiens GN=CP PE=1 SV=1 - [CERU_HUMAN] | 163.03 | 32.86% | 33 |
| P00751 | Complement factor B OS=Homo sapiens GN=CFB PE=1 SV=2 - [CFAB_HUMAN] | 16.89 | 12.17% | 7 |
| P08603 | Complement factor H OS=Homo sapiens GN=CFH PE=1 SV=4 - [CFAH_HUMAN] | 218.84 | 51.26% | 55 |
| P80200 | Cytotoxicity-associated immunodominant antigen OS=Helicobacter pylori GN=cagA PE=1 SV=1 - [CGA1_HELPX] | 18.85 | 6.10% | 2 |
| P55746 | Cytotoxicity-associated immunodominant antigen OS=Helicobacter pylori GN=cagA PE=4 SV=1 - [CGA2_HELPX] | 17.33 | 5.08% | 1 |
| P06276 | Cholinesterase OS=Homo sapiens GN=BCHE PE=1 SV=1 - [CHLE_HUMAN] | 7.1 | 4.82% | 3 |
| Q00610 | Clathrin heavy chain 1 OS=Homo sapiens GN=CLTC PE=1 SV=5 - [CLH1_HUMAN] | 185.62 | 33.85% | 52 |
| O00299 | Chloride intracellular channel protein 1 OS=Homo sapiens GN=CLIC1 PE=1 SV=4 - [CLIC1_HUMAN] | 7.28 | 12.45% | 2 |
| P10909 | Clusterin OS=Homo sapiens GN=CLU PE=1 SV=1 - [CLUS_HUMAN] | 6.18 | 10.47% | 3 |
| Q12860 | Contactin-1 OS=Homo sapiens GN=CNTN1 PE=1 SV=1 - [CNTN1_HUMAN] | 18.6 | 9.72% | 8 |
| P02452 | Collagen alpha-1(I) chain OS=Homo sapiens GN=COL1A1 PE=1 SV=5 - [CO1A1_HUMAN] | 1.62 | 0.61% | 1 |
| P02458 | Collagen alpha-1(II) chain OS=Homo sapiens GN=COL2A1 PE=1 SV=3 - [CO2A1_HUMAN] | 5.01 | 3.56% | 3 |
| P01024 | Complement C3 OS=Homo sapiens GN=C3 PE=1 SV=2 - [CO3_HUMAN] | 385.69 | 48.23% | 84 |
| P02462 | Collagen alpha-1(IV) chain OS=Homo sapiens GN=COL4A1 PE=1 SV=3 - [CO4A1_HUMAN] | 9.63 | 1.50% | 3 |
| P0C0L4 | Complement C4-A OS=Homo sapiens GN=C4A PE=1 SV=2 - [CO4A_HUMAN] | 692.81 | 54.70% | 3 |
| P0C0L5 | Complement C4-B OS=Homo sapiens GN=C4B PE=1 SV=2 - [CO4B_HUMAN] | 682.51 | 54.70% | 2 |
| P01031 | Complement C5 OS=Homo sapiens GN=C5 PE=1 SV=4 - [CO5_HUMAN] | 6.26 | 2.09% | 3 |
| P12109 | Collagen alpha-1(VI) chain OS=Homo sapiens GN=COL6A1 PE=1 SV=3 - [CO6A1_HUMAN] | 15.79 | 5.06% | 4 |
| P07357 | Complement component C8 alpha chain OS=Homo sapiens GN=C8A PE=1 SV=2 - [CO8A_HUMAN] | 8.66 | 6.16% | 3 |
| P07358 | Complement component C8 beta chain OS=Homo sapiens GN=C8B PE=1 SV=3 - [CO8B_HUMAN] | 0 | 1.52% | 1 |
| P07360 | Complement component C8 gamma chain OS=Homo sapiens GN=C8G PE=1 SV=3 - [CO8G_HUMAN] | 4.09 | 7.43% | 1 |
| P02748 | Complement component C9 OS=Homo sapiens GN=C9 PE=1 SV=2 - [CO9_HUMAN] | 27.88 | 5.90% | 5 |
| P23528 | Cofilin-1 OS=Homo sapiens GN=CFL1 PE=1 SV=3 - [COF1_HUMAN] | 3.41 | 11.45% | 1 |
| P39060 | Collagen alpha-1(XVIII) chain OS=Homo sapiens GN=COL18A1 PE=1 SV=5 - [COIA1_HUMAN] | 3.64 | 2.34% | 2 |
| Q96P44 | Collagen alpha-1(XXI) chain OS=Homo sapiens GN=COL21A1 PE=2 SV=1 - [COLA1_HUMAN] | 2.25 | 2.19% | 1 |
| P49747 | Cartilage oligomeric matrix protein OS=Homo sapiens GN=COMP PE=1 SV=2 - [COMP_HUMAN] | 2.02 | 1.32% | 1 |
| Q9BV73 | Centrosome-associated protein CEP250 OS=Homo sapiens GN=CEP250 PE=1 SV=2 - [CP250_HUMAN] | 2.09 | 0.70% | 1 |
| Q86XI8 | Uncharacterized protein C19orf68 OS=Homo sapiens GN=C19orf68 PE=1 SV=2 - [CS068_HUMAN] | 1.91 | 1.59% | 1 |
| O94985 | Calsyntenin-1 OS=Homo sapiens GN=CLSTN1 PE=1 SV=1 - [CSTN1_HUMAN] | 12.36 | 7.75% | 6 |
| Q96CG8 | Collagen triple helix repeat-containing protein 1 OS=Homo sapiens GN=CTHRC1 PE=1 SV=1 - [CTHR1_HUMAN] | 2.04 | 3.29% | 1 |
| Q8WWI5 | Choline transporter-like protein 1 OS=Homo sapiens GN=SLC44A1 PE=1 SV=1 - [CTL1_HUMAN] | 11.54 | 7.91% | 4 |
| Q8IWA5 | Choline transporter-like protein 2 OS=Homo sapiens GN=SLC44A2 PE=1 SV=3 - [CTL2_HUMAN] | 87.31 | 28.61% | 23 |
| P35221 | Catenin alpha-1 OS=Homo sapiens GN=CTNNA1 PE=1 SV=1 - [CTNA1_HUMAN] | 100.1 | 45.92% | 30 |
| P35222 | Catenin beta-1 OS=Homo sapiens GN=CTNNB1 PE=1 SV=1 - [CTNB1_HUMAN] | 58.66 | 27.78% | 15 |
| O60716 | Catenin delta-1 OS=Homo sapiens GN=CTNND1 PE=1 SV=1 - [CTND1_HUMAN] | 36.97 | 26.65% | 18 |
| P30825 | High affinity cationic amino acid transporter 1 OS=Homo sapiens GN=SLC7A1 PE=1 SV=1 - [CTR1_HUMAN] | 8.52 | 4.93% | 3 |
| Q7L576 | Cytoplasmic FMR1-interacting protein 1 OS=Homo sapiens GN=CYFIP1 PE=1 SV=1 - [CYFP1_HUMAN] | 30.09 | 9.26% | 9 |
| Q16531 | DNA damage-binding protein 1 OS=Homo sapiens GN=DDB1 PE=1 SV=1 - [DDB1_HUMAN] | 2.1 | 2.63% | 1 |
| Q8IY21 | Probable ATP-dependent RNA helicase DDX60 OS=Homo sapiens GN=DDX60 PE=1 SV=3 - [DDX60_HUMAN] | 0 | 0.99% | 1 |
| Q9P265 | Disco-interacting protein 2 homolog B OS=Homo sapiens GN=DIP2B PE=1 SV=3 - [DIP2B_HUMAN] | 227.06 | 41.43% | 53 |
| Q9Y2E4 | Disco-interacting protein 2 homolog C OS=Homo sapiens GN=DIP2C PE=1 SV=2 - [DIP2C_HUMAN] | 12.7 | 4.56% | 3 |
| Q12959 | Disks large homolog 1 OS=Homo sapiens GN=DLG1 PE=1 SV=2 - [DLG1_HUMAN] | 26.27 | 15.49% | 10 |
| P31689 | DnaJ homolog subfamily A member 1 OS=Homo sapiens GN=DNAJA1 PE=1 SV=2 - [DNJA1_HUMAN] | 5.59 | 8.82% | 3 |
| P09172 | Dopamine beta-hydroxylase OS=Homo sapiens GN=DBH PE=1 SV=3 - [DOPO_HUMAN] | 3.04 | 1.62% | 1 |
| P27487 | Dipeptidyl peptidase 4 OS=Homo sapiens GN=DPP4 PE=1 SV=2 - [DPP4_HUMAN] | 23.01 | 10.57% | 8 |
| Q16555 | Dihydropyrimidinase-related protein 2 OS=Homo sapiens GN=DPYSL2 PE=1 SV=1 - [DPYL2_HUMAN] | 3.11 | 2.62% | 1 |
| P68104 | Elongation factor 1-alpha 1 OS=Homo sapiens GN=EEF1A1 PE=1 SV=1 - [EF1A1_HUMAN] | 20.19 | 16.45% | 6 |
| P29692 | Elongation factor 1-delta OS=Homo sapiens GN=EEF1D PE=1 SV=5 - [EF1D_HUMAN] | 2.71 | 4.27% | 1 |
| P26641 | Elongation factor 1-gamma OS=Homo sapiens GN=EEF1G PE=1 SV=3 - [EF1G_HUMAN] | 11.88 | 10.76% | 4 |
| P13639 | Elongation factor 2 OS=Homo sapiens GN=EEF2 PE=1 SV=4 - [EF2_HUMAN] | 19.03 | 15.27% | 10 |
| P00533 | Epidermal growth factor receptor OS=Homo sapiens GN=EGFR PE=1 SV=2 - [EGFR_HUMAN] | 74.65 | 26.36% | 26 |
| P17813 | Endoglin OS=Homo sapiens GN=ENG PE=1 SV=2 - [EGLN_HUMAN] | 16.17 | 8.66% | 4 |
| Q9H4M9 | EH domain-containing protein 1 OS=Homo sapiens GN=EHD1 PE=1 SV=2 - [EHD1_HUMAN] | 31.96 | 15.92% | 3 |
| Q9NZN4 | EH domain-containing protein 2 OS=Homo sapiens GN=EHD2 PE=1 SV=2 - [EHD2_HUMAN] | 2.1 | 2.03% | 1 |
| Q9NZN3 | EH domain-containing protein 3 OS=Homo sapiens GN=EHD3 PE=1 SV=2 - [EHD3_HUMAN] | 53.29 | 22.99% | 5 |
| Q9H223 | EH domain-containing protein 4 OS=Homo sapiens GN=EHD4 PE=1 SV=1 - [EHD4_HUMAN] | 22.3 | 9.98% | 3 |
| P55884 | Eukaryotic translation initiation factor 3 subunit B OS=Homo sapiens GN=EIF3B PE=1 SV=3 - [EIF3B_HUMAN] | 2.12 | 2.46% | 2 |
| P06733 | Alpha-enolase OS=Homo sapiens GN=ENO1 PE=1 SV=2 - [ENOA_HUMAN] | 18.79 | 17.74% | 6 |
| P14625 | Endoplasmin OS=Homo sapiens GN=HSP90B1 PE=1 SV=1 - [ENPL_HUMAN] | 16.79 | 9.84% | 5 |
| P16422 | Epithelial cell adhesion molecule OS=Homo sapiens GN=EPCAM PE=1 SV=2 - [EPCAM_HUMAN] | 3.54 | 7.96% | 1 |
| P29317 | Ephrin type-A receptor 2 OS=Homo sapiens GN=EPHA2 PE=1 SV=2 - [EPHA2_HUMAN] | 42.66 | 19.16% | 17 |
| P29323 | Ephrin type-B receptor 2 OS=Homo sapiens GN=EPHB2 PE=1 SV=5 - [EPHB2_HUMAN] | 5.04 | 3.32% | 1 |
| Q8TE68 | Epidermal growth factor receptor kinase substrate 8-like protein 1 OS=Homo sapiens GN=EPS8L1 PE=1 SV=3 - [ES8L1_HUMAN] | 2.61 | 3.18% | 1 |
| P15311 | Ezrin OS=Homo sapiens GN=EZR PE=1 SV=4 - [EZRI_HUMAN] | 18.35 | 12.80% | 3 |
| Q6SJ93 | Protein FAM111B OS=Homo sapiens GN=FAM111B PE=1 SV=1 - [F111B_HUMAN] | 2.7 | 1.36% | 1 |
| P00488 | Coagulation factor XIII A chain OS=Homo sapiens GN=F13A1 PE=1 SV=4 - [F13A_HUMAN] | 3.02 | 2.19% | 1 |
| P00742 | Coagulation factor X OS=Homo sapiens GN=F10 PE=1 SV=2 - [FA10_HUMAN] | 4.81 | 3.69% | 2 |
| P03951 | Coagulation factor XI OS=Homo sapiens GN=F11 PE=1 SV=1 - [FA11_HUMAN] | 17.85 | 14.72% | 8 |
| Q96MK3 | Protein FAM20A OS=Homo sapiens GN=FAM20A PE=1 SV=4 - [FA20A_HUMAN] | 3.4 | 2.22% | 1 |
| Q9NUQ9 | Protein FAM49B OS=Homo sapiens GN=FAM49B PE=1 SV=1 - [FA49B_HUMAN] | 2.4 | 2.78% | 1 |
| P12259 | Coagulation factor V OS=Homo sapiens GN=F5 PE=1 SV=4 - [FA5_HUMAN] | 31.42 | 4.32% | 8 |
| P23142 | Fibulin-1 OS=Homo sapiens GN=FBLN1 PE=1 SV=4 - [FBLN1_HUMAN] | 5.3 | 7.40% | 3 |
| Q9Y6R7 | IgGFc-binding protein OS=Homo sapiens GN=FCGBP PE=1 SV=3 - [FCGBP_HUMAN] | 12.26 | 1.52% | 6 |
| O75636 | Ficolin-3 OS=Homo sapiens GN=FCN3 PE=1 SV=2 - [FCN3_HUMAN] | 18.52 | 22.41% | 7 |
| P02765 | Alpha-2-HS-glycoprotein OS=Homo sapiens GN=AHSG PE=1 SV=1 - [FETUA_HUMAN] | 12.11 | 3.54% | 2 |
| P02675 | Fibrinogen beta chain OS=Homo sapiens GN=FGB PE=1 SV=2 - [FIBB_HUMAN] | 2.01 | 1.83% | 1 |
| P02679 | Fibrinogen gamma chain OS=Homo sapiens GN=FGG PE=1 SV=3 - [FIBG_HUMAN] | 2.54 | 3.97% | 1 |
| Q9BVA6 | Adenosine monophosphate-protein transferase FICD OS=Homo sapiens GN=FICD PE=1 SV=2 - [FICD_HUMAN] | 0 | 4.59% | 1 |
| P02751 | Fibronectin OS=Homo sapiens GN=FN1 PE=1 SV=4 - [FINC_HUMAN] | 149.09 | 24.27% | 42 |
| P21333 | Filamin-A OS=Homo sapiens GN=FLNA PE=1 SV=4 - [FLNA_HUMAN] | 19.15 | 2.49% | 4 |
| Q14254 | Flotillin-2 OS=Homo sapiens GN=FLOT2 PE=1 SV=2 - [FLOT2_HUMAN] | 1.89 | 1.87% | 1 |
| Q96PY5 | Formin-like protein 2 OS=Homo sapiens GN=FMNL2 PE=1 SV=3 - [FMNL2_HUMAN] | 11.66 | 6.35% | 5 |
| Q06828 | Fibromodulin OS=Homo sapiens GN=FMOD PE=1 SV=2 - [FMOD_HUMAN] | 0 | 2.93% | 1 |
| Q9P2B2 | Prostaglandin F2 receptor negative regulator OS=Homo sapiens GN=PTGFRN PE=1 SV=2 - [FPRP_HUMAN] | 223.65 | 49.83% | 48 |
| P02794 | Ferritin heavy chain OS=Homo sapiens GN=FTH1 PE=1 SV=2 - [FRIH_HUMAN] | 8.25 | 10.93% | 2 |
| O60353 | Frizzled-6 OS=Homo sapiens GN=FZD6 PE=1 SV=2 - [FZD6_HUMAN] | 2.13 | 2.83% | 1 |
| P04406 | Glyceraldehyde-3-phosphate dehydrogenase OS=Homo sapiens GN=GAPDH PE=1 SV=3 - [G3P_HUMAN] | 34.84 | 39.10% | 10 |
| P06744 | Glucose-6-phosphate isomerase OS=Homo sapiens GN=GPI PE=1 SV=4 - [G6PI_HUMAN] | 7.68 | 2.69% | 1 |
| P62873 | Guanine nucleotide-binding protein G(I)/G(S)/G(T) subunit beta-1 OS=Homo sapiens GN=GNB1 PE=1 SV=3 - [GBB1_HUMAN] | 5.56 | 7.35% | 2 |
| P50395 | Rab GDP dissociation inhibitor beta OS=Homo sapiens GN=GDI2 PE=1 SV=2 - [GDIB_HUMAN] | 3.09 | 2.47% | 1 |
| P07093 | Glia-derived nexin OS=Homo sapiens GN=SERPINE2 PE=1 SV=1 - [GDN_HUMAN] | 8.22 | 7.79% | 3 |
| P06396 | Gelsolin OS=Homo sapiens GN=GSN PE=1 SV=1 - [GELS_HUMAN] | 29.66 | 15.47% | 11 |
| P04899 | Guanine nucleotide-binding protein G(i) subunit alpha-2 OS=Homo sapiens GN=GNAI2 PE=1 SV=3 - [GNAI2_HUMAN] | 7.77 | 9.58% | 1 |
| P08754 | Guanine nucleotide-binding protein G(k) subunit alpha OS=Homo sapiens GN=GNAI3 PE=1 SV=3 - [GNAI3_HUMAN] | 7.33 | 9.60% | 1 |
| A6NDK9 | Putative golgin subfamily A member 6C OS=Homo sapiens GN=GOLGA6C PE=5 SV=1 - [GOG6C_HUMAN] | 1.71 | 1.73% | 1 |
| O00461 | Golgi integral membrane protein 4 OS=Homo sapiens GN=GOLIM4 PE=1 SV=1 - [GOLI4_HUMAN] | 2.46 | 1.58% | 1 |
| P13224 | Platelet glycoprotein Ib beta chain OS=Homo sapiens GN=GP1BB PE=1 SV=1 - [GP1BB_HUMAN] | 5.86 | 6.31% | 2 |
| P35052 | Glypican-1 OS=Homo sapiens GN=GPC1 PE=1 SV=2 - [GPC1_HUMAN] | 22.42 | 18.82% | 8 |
| Q9NQ84 | G-protein coupled receptor family C group 5 member C OS=Homo sapiens GN=GPRC5C PE=1 SV=2 - [GPC5C_HUMAN] | 2.43 | 3.40% | 1 |
| Q92643 | GPI-anchor transamidase OS=Homo sapiens GN=PIGK PE=1 SV=2 - [GPI8_HUMAN] | 0 | 5.82% | 1 |
| O43194 | G-protein coupled receptor 39 OS=Homo sapiens GN=GPR39 PE=1 SV=1 - [GPR39_HUMAN] | 6.48 | 8.17% | 3 |
| Q9Y653 | G-protein coupled receptor 56 OS=Homo sapiens GN=GPR56 PE=1 SV=2 - [GPR56_HUMAN] | 2.93 | 1.88% | 1 |
| P22352 | Glutathione peroxidase 3 OS=Homo sapiens GN=GPX3 PE=1 SV=2 - [GPX3_HUMAN] | 2.98 | 5.31% | 1 |
| P11021 | 78 kDa glucose-regulated protein OS=Homo sapiens GN=HSPA5 PE=1 SV=2 - [GRP78_HUMAN] | 15.96 | 7.65% | 2 |
| Q92896 | Golgi apparatus protein 1 OS=Homo sapiens GN=GLG1 PE=1 SV=2 - [GSLG1_HUMAN] | 36.71 | 13.99% | 14 |
| Q7RTV2 | Glutathione S-transferase A5 OS=Homo sapiens GN=GSTA5 PE=1 SV=1 - [GSTA5_HUMAN] | 1.72 | 3.60% | 1 |
| P28161 | Glutathione S-transferase Mu 2 OS=Homo sapiens GN=GSTM2 PE=1 SV=2 - [GSTM2_HUMAN] | 2.73 | 4.13% | 1 |
| P09211 | Glutathione S-transferase P OS=Homo sapiens GN=GSTP1 PE=1 SV=2 - [GSTP1_HUMAN] | 2.5 | 4.76% | 1 |
| P11166 | Solute carrier family 2, facilitated glucose transporter member 1 OS=Homo sapiens GN=SLC2A1 PE=1 SV=2 - [GTR1_HUMAN] | 43.08 | 13.01% | 7 |
| P11169 | Solute carrier family 2, facilitated glucose transporter member 3 OS=Homo sapiens GN=SLC2A3 PE=1 SV=1 - [GTR3_HUMAN] | 37.77 | 16.94% | 6 |
| P16403 | Histone H1.2 OS=Homo sapiens GN=HIST1H1C PE=1 SV=2 - [H12_HUMAN] | 5.35 | 9.86% | 2 |
| Q96KK5 | Histone H2A type 1-H OS=Homo sapiens GN=HIST1H2AH PE=1 SV=3 - [H2A1H_HUMAN] | 11.65 | 27.34% | 3 |
| O60814 | Histone H2B type 1-K OS=Homo sapiens GN=HIST1H2BK PE=1 SV=3 - [H2B1K_HUMAN] | 6.25 | 14.29% | 2 |
| P68431 | Histone H3.1 OS=Homo sapiens GN=HIST1H3A PE=1 SV=2 - [H31_HUMAN] | 1.95 | 13.24% | 2 |
| P62805 | Histone H4 OS=Homo sapiens GN=HIST1H4A PE=1 SV=2 - [H4_HUMAN] | 11.01 | 31.07% | 3 |
| Q14520 | Hyaluronan-binding protein 2 OS=Homo sapiens GN=HABP2 PE=1 SV=1 - [HABP2_HUMAN] | 6.22 | 4.82% | 4 |
| P69905 | Hemoglobin subunit alpha OS=Homo sapiens GN=HBA1 PE=1 SV=2 - [HBA_HUMAN] | 27.96 | 34.51% | 4 |
| P02042 | Hemoglobin subunit delta OS=Homo sapiens GN=HBD PE=1 SV=2 - [HBD_HUMAN] | 17.79 | 6.80% | 1 |
| P02790 | Hemopexin OS=Homo sapiens GN=HPX PE=1 SV=2 - [HEMO_HUMAN] | 2.98 | 2.38% | 1 |
| P05546 | Heparin cofactor 2 OS=Homo sapiens GN=SERPIND1 PE=1 SV=3 - [HEP2_HUMAN] | 2.56 | 5.21% | 2 |
| O14964 | Hepatocyte growth factor-regulated tyrosine kinase substrate OS=Homo sapiens GN=HGS PE=1 SV=1 - [HGS_HUMAN] | 15.23 | 9.52% | 7 |
| Q00839 | Heterogeneous nuclear ribonucleoprotein U OS=Homo sapiens GN=HNRNPU PE=1 SV=6 - [HNRPU_HUMAN] | 3.77 | 1.82% | 1 |
| P00739 | Haptoglobin-related protein OS=Homo sapiens GN=HPR PE=2 SV=2 - [HPTR_HUMAN] | 38.92 | 49.14% | 6 |
| P00738 | Haptoglobin OS=Homo sapiens GN=HP PE=1 SV=1 - [HPT_HUMAN] | 35.65 | 29.80% | 4 |
| P04196 | Histidine-rich glycoprotein OS=Homo sapiens GN=HRG PE=1 SV=1 - [HRG_HUMAN] | 12.14 | 10.29% | 4 |
| P07900 | Heat shock protein HSP 90-alpha OS=Homo sapiens GN=HSP90AA1 PE=1 SV=5 - [HS90A_HUMAN] | 52.01 | 23.63% | 11 |
| P08238 | Heat shock protein HSP 90-beta OS=Homo sapiens GN=HSP90AB1 PE=1 SV=4 - [HS90B_HUMAN] | 48.79 | 18.92% | 6 |
| P48723 | Heat shock 70 kDa protein 13 OS=Homo sapiens GN=HSPA13 PE=1 SV=1 - [HSP13_HUMAN] | 4.26 | 3.61% | 2 |
| P08107 | Heat shock 70 kDa protein 1A/1B OS=Homo sapiens GN=HSPA1A PE=1 SV=5 - [HSP71_HUMAN] | 54.86 | 28.39% | 13 |
| P34932 | Heat shock 70 kDa protein 4 OS=Homo sapiens GN=HSPA4 PE=1 SV=4 - [HSP74_HUMAN] | 6.69 | 5.12% | 3 |
| P11142 | Heat shock cognate 71 kDa protein OS=Homo sapiens GN=HSPA8 PE=1 SV=1 - [HSP7C_HUMAN] | 124.01 | 45.82% | 22 |
| P04792 | Heat shock protein beta-1 OS=Homo sapiens GN=HSPB1 PE=1 SV=2 - [HSPB1_HUMAN] | 3.19 | 4.88% | 1 |
| Q92743 | Serine protease HTRA1 OS=Homo sapiens GN=HTRA1 PE=1 SV=1 - [HTRA1_HUMAN] | 2.89 | 2.92% | 1 |
| P01743 | Ig heavy chain V-I region HG3 OS=Homo sapiens PE=3 SV=1 - [HV102_HUMAN] | 3.23 | 9.40% | 1 |
| P23083 | Ig heavy chain V-I region V35 OS=Homo sapiens PE=1 SV=1 - [HV103_HUMAN] | 1.68 | 10.26% | 1 |
| P01825 | Ig heavy chain V-II region NEWM OS=Homo sapiens PE=1 SV=1 - [HV207_HUMAN] | 3.06 | 13.68% | 1 |
| P04438 | Ig heavy chain V-II region SESS OS=Homo sapiens PE=2 SV=1 - [HV208_HUMAN] | 5.26 | 10.88% | 2 |
| P01763 | Ig heavy chain V-III region WEA OS=Homo sapiens PE=1 SV=1 - [HV302_HUMAN] | 7.62 | 26.32% | 1 |
| P01764 | Ig heavy chain V-III region VH26 OS=Homo sapiens PE=1 SV=1 - [HV303_HUMAN] | 7.29 | 18.80% | 1 |
| P01765 | Ig heavy chain V-III region TIL OS=Homo sapiens PE=1 SV=1 - [HV304_HUMAN] | 11.66 | 26.09% | 1 |
| P01766 | Ig heavy chain V-III region BRO OS=Homo sapiens PE=1 SV=1 - [HV305_HUMAN] | 18.21 | 25.00% | 1 |
| P01781 | Ig heavy chain V-III region GAL OS=Homo sapiens PE=1 SV=1 - [HV320_HUMAN] | 13.82 | 31.03% | 3 |
| Q9Y4L1 | Hypoxia up-regulated protein 1 OS=Homo sapiens GN=HYOU1 PE=1 SV=1 - [HYOU1_HUMAN] | 2.14 | 1.30% | 1 |
| P05155 | Plasma protease C1 inhibitor OS=Homo sapiens GN=SERPING1 PE=1 SV=2 - [IC1_HUMAN] | 6.93 | 5.40% | 3 |
| P05362 | Intercellular adhesion molecule 1 OS=Homo sapiens GN=ICAM1 PE=1 SV=2 - [ICAM1_HUMAN] | 12.78 | 14.66% | 5 |
| P60842 | Eukaryotic initiation factor 4A-I OS=Homo sapiens GN=EIF4A1 PE=1 SV=1 - [IF4A1_HUMAN] | 2.9 | 2.46% | 1 |
| P01876 | Ig alpha-1 chain C region OS=Homo sapiens GN=IGHA1 PE=1 SV=2 - [IGHA1_HUMAN] | 154.47 | 57.79% | 6 |
| P01877 | Ig alpha-2 chain C region OS=Homo sapiens GN=IGHA2 PE=1 SV=3 - [IGHA2_HUMAN] | 105.68 | 54.41% | 3 |
| P01857 | Ig gamma-1 chain C region OS=Homo sapiens GN=IGHG1 PE=1 SV=1 - [IGHG1_HUMAN] | 135.75 | 66.36% | 9 |
| P01859 | Ig gamma-2 chain C region OS=Homo sapiens GN=IGHG2 PE=1 SV=2 - [IGHG2_HUMAN] | 108.62 | 53.37% | 7 |
| P01860 | Ig gamma-3 chain C region OS=Homo sapiens GN=IGHG3 PE=1 SV=2 - [IGHG3_HUMAN] | 68.21 | 44.03% | 4 |
| P01861 | Ig gamma-4 chain C region OS=Homo sapiens GN=IGHG4 PE=1 SV=1 - [IGHG4_HUMAN] | 69.06 | 48.32% | 6 |
| P01871 | Ig mu chain C region OS=Homo sapiens GN=IGHM PE=1 SV=3 - [IGHM_HUMAN] | 28.7 | 18.81% | 6 |
| P01591 | Immunoglobulin J chain OS=Homo sapiens GN=IGJ PE=1 SV=4 - [IGJ_HUMAN] | 17.02 | 26.42% | 6 |
| P01834 | Ig kappa chain C region OS=Homo sapiens GN=IGKC PE=1 SV=1 - [IGKC_HUMAN] | 78.27 | 97.17% | 9 |
| B9A064 | Immunoglobulin lambda-like polypeptide 5 OS=Homo sapiens GN=IGLL5 PE=2 SV=2 - [IGLL5_HUMAN] | 46.13 | 40.65% | 4 |
| P12268 | Inosine-5'-monophosphate dehydrogenase 2 OS=Homo sapiens GN=IMPDH2 PE=1 SV=2 - [IMDH2_HUMAN] | 19.4 | 15.56% | 7 |
| P15260 | Interferon gamma receptor 1 OS=Homo sapiens GN=IFNGR1 PE=1 SV=1 - [INGR1_HUMAN] | 2.28 | 2.25% | 1 |
| Q96P70 | Importin-9 OS=Homo sapiens GN=IPO9 PE=1 SV=3 - [IPO9_HUMAN] | 2.01 | 0.86% | 1 |
| P05154 | Plasma serine protease inhibitor OS=Homo sapiens GN=SERPINA5 PE=1 SV=3 - [IPSP_HUMAN] | 28.36 | 19.46% | 9 |
| P46940 | Ras GTPase-activating-like protein IQGAP1 OS=Homo sapiens GN=IQGAP1 PE=1 SV=1 - [IQGA1_HUMAN] | 24.05 | 5.49% | 7 |
| P53990 | IST1 homolog OS=Homo sapiens GN=IST1 PE=1 SV=1 - [IST1_HUMAN] | 4.55 | 4.67% | 2 |
| P08514 | Integrin alpha-IIb OS=Homo sapiens GN=ITGA2B PE=1 SV=3 - [ITA2B_HUMAN] | 41.6 | 14.53% | 13 |
| P17301 | Integrin alpha-2 OS=Homo sapiens GN=ITGA2 PE=1 SV=1 - [ITA2_HUMAN] | 148.27 | 35.56% | 33 |
| P26006 | Integrin alpha-3 OS=Homo sapiens GN=ITGA3 PE=1 SV=5 - [ITA3_HUMAN] | 81.53 | 20.74% | 21 |
| P08648 | Integrin alpha-5 OS=Homo sapiens GN=ITGA5 PE=1 SV=2 - [ITA5_HUMAN] | 35.42 | 11.25% | 11 |
| P11215 | Integrin alpha-M OS=Homo sapiens GN=ITGAM PE=1 SV=2 - [ITAM_HUMAN] | 2.98 | 0.87% | 1 |
| P06756 | Integrin alpha-V OS=Homo sapiens GN=ITGAV PE=1 SV=2 - [ITAV_HUMAN] | 73.95 | 32.06% | 25 |
| P05556 | Integrin beta-1 OS=Homo sapiens GN=ITGB1 PE=1 SV=2 - [ITB1_HUMAN] | 161.47 | 44.11% | 30 |
| P05106 | Integrin beta-3 OS=Homo sapiens GN=ITGB3 PE=1 SV=2 - [ITB3_HUMAN] | 8.8 | 6.60% | 4 |
| P16144 | Integrin beta-4 OS=Homo sapiens GN=ITGB4 PE=1 SV=5 - [ITB4_HUMAN] | 123.81 | 30.24% | 46 |
| P18084 | Integrin beta-5 OS=Homo sapiens GN=ITGB5 PE=1 SV=1 - [ITB5_HUMAN] | 41.54 | 18.52% | 11 |
| P26012 | Integrin beta-8 OS=Homo sapiens GN=ITGB8 PE=1 SV=1 - [ITB8_HUMAN] | 2.35 | 1.43% | 1 |
| Q96J02 | E3 ubiquitin-protein ligase Itchy homolog OS=Homo sapiens GN=ITCH PE=1 SV=2 - [ITCH_HUMAN] | 0 | 1.44% | 1 |
| P19827 | Inter-alpha-trypsin inhibitor heavy chain H1 OS=Homo sapiens GN=ITIH1 PE=1 SV=3 - [ITIH1_HUMAN] | 12.9 | 6.48% | 5 |
| P19823 | Inter-alpha-trypsin inhibitor heavy chain H2 OS=Homo sapiens GN=ITIH2 PE=1 SV=2 - [ITIH2_HUMAN] | 45.93 | 17.02% | 15 |
| Q06033 | Inter-alpha-trypsin inhibitor heavy chain H3 OS=Homo sapiens GN=ITIH3 PE=1 SV=2 - [ITIH3_HUMAN] | 20.59 | 8.31% | 7 |
| Q14624 | Inter-alpha-trypsin inhibitor heavy chain H4 OS=Homo sapiens GN=ITIH4 PE=1 SV=4 - [ITIH4_HUMAN] | 30.68 | 9.78% | 8 |
| Q9Y287 | Integral membrane protein 2B OS=Homo sapiens GN=ITM2B PE=1 SV=1 - [ITM2B_HUMAN] | 0 | 10.90% | 1 |
| Q7Z3Z0 | Keratin, type I cytoskeletal 25 OS=Homo sapiens GN=KRT25 PE=1 SV=1 - [K1C25_HUMAN] | 2.3 | 2.00% | 1 |
| P04264 | Keratin, type II cytoskeletal 1 OS=Homo sapiens GN=KRT1 PE=1 SV=6 - [K2C1_HUMAN] | 10.12 | 5.90% | 3 |
| P17858 | ATP-dependent 6-phosphofructokinase, liver type OS=Homo sapiens GN=PFKL PE=1 SV=6 - [PFKAL_HUMAN] | 2.13 | 2.18% | 1 |
| Q14678 | KN motif and ankyrin repeat domain-containing protein 1 OS=Homo sapiens GN=KANK1 PE=1 SV=3 - [KANK1_HUMAN] | 2.54 | 1.33% | 1 |
| Q02241 | Kinesin-like protein KIF23 OS=Homo sapiens GN=KIF23 PE=1 SV=3 - [KIF23_HUMAN] | 3.66 | 1.35% | 1 |
| P10721 | Mast/stem cell growth factor receptor Kit OS=Homo sapiens GN=KIT PE=1 SV=1 - [KIT_HUMAN] | 2.35 | 1.02% | 1 |
| P03952 | Plasma kallikrein OS=Homo sapiens GN=KLKB1 PE=1 SV=1 - [KLKB1_HUMAN] | 47.91 | 30.72% | 17 |
| P01042 | Kininogen-1 OS=Homo sapiens GN=KNG1 PE=1 SV=2 - [KNG1_HUMAN] | 2.06 | 2.02% | 1 |
| P14618 | Pyruvate kinase PKM OS=Homo sapiens GN=PKM PE=1 SV=4 - [KPYM_HUMAN] | 43.26 | 32.39% | 15 |
| P01593 | Ig kappa chain V-I region AG OS=Homo sapiens PE=1 SV=1 - [KV101_HUMAN] | 5.21 | 27.78% | 2 |
| P01597 | Ig kappa chain V-I region DEE OS=Homo sapiens PE=1 SV=1 - [KV105_HUMAN] | 9.86 | 16.67% | 1 |
| P01598 | Ig kappa chain V-I region EU OS=Homo sapiens PE=1 SV=1 - [KV106_HUMAN] | 12.73 | 26.85% | 2 |
| P01605 | Ig kappa chain V-I region Lay OS=Homo sapiens PE=1 SV=1 - [KV113_HUMAN] | 2.11 | 8.33% | 1 |
| P01611 | Ig kappa chain V-I region Wes OS=Homo sapiens PE=1 SV=1 - [KV119_HUMAN] | 3.55 | 16.67% | 1 |
| P01617 | Ig kappa chain V-II region TEW OS=Homo sapiens PE=1 SV=1 - [KV204_HUMAN] | 6.17 | 38.94% | 2 |
| P06310 | Ig kappa chain V-II region RPMI 6410 OS=Homo sapiens PE=4 SV=1 - [KV206_HUMAN] | 0 | 15.04% | 1 |
| P01623 | Ig kappa chain V-III region WOL OS=Homo sapiens PE=1 SV=1 - [KV305_HUMAN] | 20 | 35.78% | 1 |
| P04433 | Ig kappa chain V-III region VG (Fragment) OS=Homo sapiens PE=1 SV=1 - [KV309_HUMAN] | 2.51 | 7.83% | 1 |
| P18135 | Ig kappa chain V-III region HAH OS=Homo sapiens PE=2 SV=1 - [KV312_HUMAN] | 11.06 | 33.33% | 1 |
| P01625 | Ig kappa chain V-IV region Len OS=Homo sapiens PE=1 SV=2 - [KV402_HUMAN] | 9.19 | 23.68% | 2 |
| P0CG05 | Ig lambda-2 chain C regions OS=Homo sapiens GN=IGLC2 PE=1 SV=1 - [LAC2_HUMAN] | 67.24 | 74.53% | 2 |
| P0CG06 | Ig lambda-3 chain C regions OS=Homo sapiens GN=IGLC3 PE=1 SV=1 - [LAC3_HUMAN] | 62.55 | 70.75% | 1 |
| A0M8Q6 | Ig lambda-7 chain C region OS=Homo sapiens GN=IGLC7 PE=1 SV=2 - [LAC7_HUMAN] | 34.87 | 46.23% | 1 |
| P11279 | Lysosome-associated membrane glycoprotein 1 OS=Homo sapiens GN=LAMP1 PE=1 SV=3 - [LAMP1_HUMAN] | 12.1 | 11.75% | 4 |
| P13473 | Lysosome-associated membrane glycoprotein 2 OS=Homo sapiens GN=LAMP2 PE=1 SV=2 - [LAMP2_HUMAN] | 2.59 | 1.95% | 1 |
| Q01650 | Large neutral amino acids transporter small subunit 1 OS=Homo sapiens GN=SLC7A5 PE=1 SV=2 - [LAT1_HUMAN] | 16.31 | 9.86% | 4 |
| Q9UIQ6 | Leucyl-cystinyl aminopeptidase OS=Homo sapiens GN=LNPEP PE=1 SV=3 - [LCAP_HUMAN] | 2.44 | 1.46% | 1 |
| P00338 | L-lactate dehydrogenase A chain OS=Homo sapiens GN=LDHA PE=1 SV=2 - [LDHA_HUMAN] | 5.55 | 6.33% | 1 |
| P07195 | L-lactate dehydrogenase B chain OS=Homo sapiens GN=LDHB PE=1 SV=2 - [LDHB_HUMAN] | 11.32 | 13.77% | 3 |
| Q969X1 | Protein lifeguard 3 OS=Homo sapiens GN=TMBIM1 PE=1 SV=2 - [LFG3_HUMAN] | 2.92 | 4.50% | 1 |
| Q08380 | Galectin-3-binding protein OS=Homo sapiens GN=LGALS3BP PE=1 SV=1 - [LG3BP_HUMAN] | 64.46 | 33.16% | 17 |
| Q9UHB6 | LIM domain and actin-binding protein 1 OS=Homo sapiens GN=LIMA1 PE=1 SV=1 - [LIMA1_HUMAN] | 0 | 1.98% | 1 |
| Q9NUN5 | Probable lysosomal cobalamin transporter OS=Homo sapiens GN=LMBRD1 PE=1 SV=1 - [LMBD1_HUMAN] | 3.13 | 3.33% | 1 |
| O95490 | Latrophilin-2 OS=Homo sapiens GN=LPHN2 PE=1 SV=2 - [LPHN2_HUMAN] | 4.23 | 1.51% | 1 |
| Q07954 | Prolow-density lipoprotein receptor-related protein 1 OS=Homo sapiens GN=LRP1 PE=1 SV=2 - [LRP1_HUMAN] | 38.59 | 2.97% | 10 |
| Q86X29 | Lipolysis-stimulated lipoprotein receptor OS=Homo sapiens GN=LSR PE=1 SV=4 - [LSR_HUMAN] | 13.02 | 15.25% | 6 |
| P51884 | Lumican OS=Homo sapiens GN=LUM PE=1 SV=2 - [LUM_HUMAN] | 13.5 | 11.24% | 5 |
| P01700 | Ig lambda chain V-I region HA OS=Homo sapiens PE=1 SV=1 - [LV102_HUMAN] | 5.6 | 23.21% | 2 |
| P01705 | Ig lambda chain V-II region NEI OS=Homo sapiens PE=1 SV=1 - [LV202_HUMAN] | 2.6 | 7.21% | 1 |
| P01714 | Ig lambda chain V-III region SH OS=Homo sapiens PE=1 SV=1 - [LV301_HUMAN] | 7.02 | 16.67% | 1 |
| P80748 | Ig lambda chain V-III region LOI OS=Homo sapiens PE=1 SV=1 - [LV302_HUMAN] | 10.67 | 37.84% | 3 |
| P01717 | Ig lambda chain V-IV region Hil OS=Homo sapiens PE=1 SV=1 - [LV403_HUMAN] | 5.45 | 17.76% | 1 |
| P07948 | Tyrosine-protein kinase Lyn OS=Homo sapiens GN=LYN PE=1 SV=3 - [LYN_HUMAN] | 5 | 3.13% | 2 |
| O95274 | Ly6/PLAUR domain-containing protein 3 OS=Homo sapiens GN=LYPD3 PE=1 SV=2 - [LYPD3_HUMAN] | 0 | 4.05% | 1 |
| O95819 | Mitogen-activated protein kinase kinase kinase kinase 4 OS=Homo sapiens GN=MAP4K4 PE=1 SV=2 - [M4K4_HUMAN] | 90.59 | 24.54% | 16 |
| Q9UKM7 | Endoplasmic reticulum mannosyl-oligosaccharide 1,2-alpha-mannosidase OS=Homo sapiens GN=MAN1B1 PE=1 SV=2 - [MA1B1_HUMAN] | 2.78 | 3.15% | 1 |
| O00754 | Lysosomal alpha-mannosidase OS=Homo sapiens GN=MAN2B1 PE=1 SV=3 - [MA2B1_HUMAN] | 0 | 1.19% | 1 |
| Q96A59 | MARVEL domain-containing protein 3 OS=Homo sapiens GN=MARVELD3 PE=2 SV=3 - [MALD3_HUMAN] | 2.96 | 2.74% | 1 |
| P29966 | Myristoylated alanine-rich C-kinase substrate OS=Homo sapiens GN=MARCKS PE=1 SV=4 - [MARCS_HUMAN] | 2.76 | 4.52% | 1 |
| Q15555 | Microtubule-associated protein RP/EB family member 2 OS=Homo sapiens GN=MAPRE2 PE=1 SV=1 - [MARE2_HUMAN] | 20.56 | 20.49% | 5 |
| P48740 | Mannan-binding lectin serine protease 1 OS=Homo sapiens GN=MASP1 PE=1 SV=3 - [MASP1_HUMAN] | 37.46 | 18.03% | 10 |
| O00187 | Mannan-binding lectin serine protease 2 OS=Homo sapiens GN=MASP2 PE=1 SV=4 - [MASP2_HUMAN] | 2.17 | 1.17% | 1 |
| Q96EY5 | Multivesicular body subunit 12A OS=Homo sapiens GN=MVB12A PE=1 SV=1 - [MB12A_HUMAN] | 7.1 | 6.59% | 1 |
| Q8WWY6 | Methyl-CpG-binding domain protein 3-like 1 OS=Homo sapiens GN=MBD3L1 PE=2 SV=2 - [MB3L1_HUMAN] | 2.76 | 5.15% | 1 |
| O75586 | Mediator of RNA polymerase II transcription subunit 6 OS=Homo sapiens GN=MED6 PE=1 SV=2 - [MED6_HUMAN] | 2.07 | 2.85% | 1 |
| P31153 | S-adenosylmethionine synthase isoform type-2 OS=Homo sapiens GN=MAT2A PE=1 SV=1 - [METK2_HUMAN] | 0 | 3.80% | 1 |
| Q08431 | Lactadherin OS=Homo sapiens GN=MFGE8 PE=1 SV=2 - [MFGM_HUMAN] | 2.49 | 2.58% | 1 |
| P20774 | Mimecan OS=Homo sapiens GN=OGN PE=1 SV=1 - [MIME_HUMAN] | 4.68 | 4.36% | 2 |
| Q8N4C8 | Misshapen-like kinase 1 OS=Homo sapiens GN=MINK1 PE=1 SV=2 - [MINK1_HUMAN] | 105.83 | 26.43% | 22 |
| Q70IA6 | MOB kinase activator 2 OS=Homo sapiens GN=MOB2 PE=1 SV=1 - [MOB2_HUMAN] | 1.98 | 2.95% | 1 |
| O95396 | Adenylyltransferase and sulfurtransferase MOCS3 OS=Homo sapiens GN=MOCS3 PE=1 SV=1 - [MOCS3_HUMAN] | 2.99 | 2.17% | 1 |
| P26038 | Moesin OS=Homo sapiens GN=MSN PE=1 SV=3 - [MOES_HUMAN] | 16.07 | 9.88% | 2 |
| P53985 | Monocarboxylate transporter 1 OS=Homo sapiens GN=SLC16A1 PE=1 SV=3 - [MOT1_HUMAN] | 0 | 4.20% | 1 |
| P11717 | Cation-independent mannose-6-phosphate receptor OS=Homo sapiens GN=IGF2R PE=1 SV=3 - [MPRI_HUMAN] | 20.63 | 2.81% | 7 |
| Q9UBG0 | C-type mannose receptor 2 OS=Homo sapiens GN=MRC2 PE=1 SV=2 - [MRC2_HUMAN] | 5.38 | 3.31% | 4 |
| P33527 | Multidrug resistance-associated protein 1 OS=Homo sapiens GN=ABCC1 PE=1 SV=3 - [MRP1_HUMAN] | 6.04 | 1.18% | 2 |
| Q14764 | Major vault protein OS=Homo sapiens GN=MVP PE=1 SV=4 - [MVP_HUMAN] | 40.1 | 24.30% | 14 |
| P35579 | Myosin-9 OS=Homo sapiens GN=MYH9 PE=1 SV=4 - [MYH9_HUMAN] | 2.51 | 0.82% | 1 |
| P60660 | Myosin light polypeptide 6 OS=Homo sapiens GN=MYL6 PE=1 SV=2 - [MYL6_HUMAN] | 3.35 | 8.61% | 1 |
| O00159 | Unconventional myosin-Ic OS=Homo sapiens GN=MYO1C PE=1 SV=4 - [MYO1C_HUMAN] | 11.81 | 3.29% | 3 |
| Q9NQX4 | Unconventional myosin-Vc OS=Homo sapiens GN=MYO5C PE=1 SV=2 - [MYO5C_HUMAN] | 0 | 0.98% | 1 |
| Q9NZM1 | Myoferlin OS=Homo sapiens GN=MYOF PE=1 SV=1 - [MYOF_HUMAN] | 5.18 | 2.81% | 4 |
| A2RRP1 | Neuroblastoma-amplified sequence OS=Homo sapiens GN=NBAS PE=1 SV=2 - [NBAS_HUMAN] | 0 | 0.67% | 1 |
| Q9Y2A7 | Nck-associated protein 1 OS=Homo sapiens GN=NCKAP1 PE=1 SV=1 - [NCKP1_HUMAN] | 13.82 | 5.85% | 6 |
| Q96SB3 | Neurabin-2 OS=Homo sapiens GN=PPP1R9B PE=1 SV=2 - [NEB2_HUMAN] | 1.77 | 1.10% | 1 |
| Q96PU5 | E3 ubiquitin-protein ligase NEDD4-like OS=Homo sapiens GN=NEDD4L PE=1 SV=2 - [NED4L_HUMAN] | 13.7 | 3.79% | 2 |
| P46934 | E3 ubiquitin-protein ligase NEDD4 OS=Homo sapiens GN=NEDD4 PE=1 SV=4 - [NEDD4_HUMAN] | 5.75 | 3.34% | 2 |
| Q92859 | Neogenin OS=Homo sapiens GN=NEO1 PE=1 SV=2 - [NEO1_HUMAN] | 12.78 | 4.79% | 5 |
| O95631 | Netrin-1 OS=Homo sapiens GN=NTN1 PE=1 SV=2 - [NET1_HUMAN] | 4.03 | 6.29% | 3 |
| Q92542 | Nicastrin OS=Homo sapiens GN=NCSTN PE=1 SV=2 - [NICA_HUMAN] | 12.05 | 5.92% | 4 |
| P14543 | Nidogen-1 OS=Homo sapiens GN=NID1 PE=1 SV=3 - [NID1_HUMAN] | 15.88 | 3.37% | 5 |
| Q14112 | Nidogen-2 OS=Homo sapiens GN=NID2 PE=1 SV=3 - [NID2_HUMAN] | 13.45 | 2.69% | 3 |
| P55209 | Nucleosome assembly protein 1-like 1 OS=Homo sapiens GN=NAP1L1 PE=1 SV=1 - [NP1L1_HUMAN] | 4.29 | 6.91% | 2 |
| Q9Y639 | Neuroplastin OS=Homo sapiens GN=NPTN PE=1 SV=2 - [NPTN_HUMAN] | 0 | 4.27% | 1 |
| O14786 | Neuropilin-1 OS=Homo sapiens GN=NRP1 PE=1 SV=3 - [NRP1_HUMAN] | 7.02 | 4.01% | 2 |
| P30989 | Neurotensin receptor type 1 OS=Homo sapiens GN=NTSR1 PE=1 SV=2 - [NTR1_HUMAN] | 3.49 | 4.07% | 1 |
| P19338 | Nucleolin OS=Homo sapiens GN=NCL PE=1 SV=3 - [NUCL_HUMAN] | 3.26 | 1.55% | 1 |
| P0C024 | Peroxisomal coenzyme A diphosphatase NUDT7 OS=Homo sapiens GN=NUDT7 PE=2 SV=1 - [NUDT7_HUMAN] | 0 | 5.88% | 1 |
| Q9NTK5 | Obg-like ATPase 1 OS=Homo sapiens GN=OLA1 PE=1 SV=2 - [OLA1_HUMAN] | 2.13 | 3.79% | 1 |
| Q9NRN5 | Olfactomedin-like protein 3 OS=Homo sapiens GN=OLFML3 PE=2 SV=1 - [OLFL3_HUMAN] | 5.55 | 4.43% | 2 |
| Q9NQN1 | Olfactory receptor 2S2 OS=Homo sapiens GN=OR2S2 PE=2 SV=2 - [OR2S1_HUMAN] | 0 | 4.39% | 1 |
| P13674 | Prolyl 4-hydroxylase subunit alpha-1 OS=Homo sapiens GN=P4HA1 PE=1 SV=2 - [P4HA1_HUMAN] | 2.96 | 2.06% | 1 |
| Q9HBI1 | Beta-parvin OS=Homo sapiens GN=PARVB PE=1 SV=1 - [PARVB_HUMAN] | 13.69 | 9.34% | 3 |
| Q08174 | Protocadherin-1 OS=Homo sapiens GN=PCDH1 PE=1 SV=2 - [PCDH1_HUMAN] | 2.72 | 1.89% | 1 |
| Q8WUM4 | Programmed cell death 6-interacting protein OS=Homo sapiens GN=PDCD6IP PE=1 SV=1 - [PDC6I_HUMAN] | 204.74 | 68.89% | 59 |
| P30101 | Protein disulfide-isomerase A3 OS=Homo sapiens GN=PDIA3 PE=1 SV=4 - [PDIA3_HUMAN] | 78.27 | 49.50% | 23 |
| P36955 | Pigment epithelium-derived factor OS=Homo sapiens GN=SERPINF1 PE=1 SV=4 - [PEDF_HUMAN] | 20.25 | 15.55% | 5 |
| P05164 | Myeloperoxidase OS=Homo sapiens GN=MPO PE=1 SV=1 - [PERM_HUMAN] | 12.58 | 6.04% | 4 |
| P50542 | Peroxisomal targeting signal 1 receptor OS=Homo sapiens GN=PEX5 PE=1 SV=3 - [PEX5_HUMAN] | 2.19 | 2.03% | 1 |
| P98160 | Basement membrane-specific heparan sulfate proteoglycan core protein OS=Homo sapiens GN=HSPG2 PE=1 SV=4 - [PGBM_HUMAN] | 5.24 | 0.98% | 3 |
| P00558 | Phosphoglycerate kinase 1 OS=Homo sapiens GN=PGK1 PE=1 SV=3 - [PGK1_HUMAN] | 18.43 | 26.38% | 7 |
| P36871 | Phosphoglucomutase-1 OS=Homo sapiens GN=PGM1 PE=1 SV=3 - [PGM1_HUMAN] | 3.81 | 2.14% | 1 |
| P07585 | Decorin OS=Homo sapiens GN=DCN PE=1 SV=1 - [PGS2_HUMAN] | 6.25 | 3.62% | 1 |
| Q86UU1 | Pleckstrin homology-like domain family B member 1 OS=Homo sapiens GN=PHLDB1 PE=1 SV=1 - [PHLB1_HUMAN] | 2.3 | 0.65% | 1 |
| P48426 | Phosphatidylinositol 5-phosphate 4-kinase type-2 alpha OS=Homo sapiens GN=PIP4K2A PE=1 SV=2 - [PI42A_HUMAN] | 8.17 | 7.39% | 3 |
| P98161 | Polycystin-1 OS=Homo sapiens GN=PKD1 PE=1 SV=3 - [PKD1_HUMAN] | 2.58 | 0.28% | 1 |
| Q16513 | Serine/threonine-protein kinase N2 OS=Homo sapiens GN=PKN2 PE=1 SV=1 - [PKN2_HUMAN] | 3.2 | 2.74% | 1 |
| Q9Y446 | Plakophilin-3 OS=Homo sapiens GN=PKP3 PE=1 SV=1 - [PKP3_HUMAN] | 19.44 | 12.80% | 8 |
| Q99569 | Plakophilin-4 OS=Homo sapiens GN=PKP4 PE=1 SV=2 - [PKP4_HUMAN] | 2.3 | 0.84% | 1 |
| P14923 | Junction plakoglobin OS=Homo sapiens GN=JUP PE=1 SV=3 - [PLAK_HUMAN] | 20.39 | 12.35% | 6 |
| Q13393 | Phospholipase D1 OS=Homo sapiens GN=PLD1 PE=1 SV=1 - [PLD1_HUMAN] | 0 | 1.77% | 1 |
| P08567 | Pleckstrin OS=Homo sapiens GN=PLEK PE=1 SV=3 - [PLEK_HUMAN] | 3.18 | 4.00% | 1 |
| P00747 | Plasminogen OS=Homo sapiens GN=PLG PE=1 SV=2 - [PLMN_HUMAN] | 0 | 2.84% | 1 |
| Q9NRY6 | Phospholipid scramblase 3 OS=Homo sapiens GN=PLSCR3 PE=1 SV=2 - [PLS3_HUMAN] | 3.36 | 6.44% | 1 |
| P55058 | Phospholipid transfer protein OS=Homo sapiens GN=PLTP PE=1 SV=1 - [PLTP_HUMAN] | 4.24 | 4.46% | 2 |
| O15031 | Plexin-B2 OS=Homo sapiens GN=PLXNB2 PE=1 SV=3 - [PLXB2_HUMAN] | 22.6 | 5.22% | 8 |
| O00592 | Podocalyxin OS=Homo sapiens GN=PODXL PE=1 SV=2 - [PODXL_HUMAN] | 2.91 | 2.15% | 1 |
| P62937 | Peptidyl-prolyl cis-trans isomerase A OS=Homo sapiens GN=PPIA PE=1 SV=2 - [PPIA_HUMAN] | 2.41 | 7.27% | 1 |
| P07737 | Profilin-1 OS=Homo sapiens GN=PFN1 PE=1 SV=2 - [PROF1_HUMAN] | 3.89 | 14.29% | 2 |
| Q8N271 | Prominin-2 OS=Homo sapiens GN=PROM2 PE=1 SV=1 - [PROM2_HUMAN] | 63.32 | 26.50% | 19 |
| P07225 | Vitamin K-dependent protein S OS=Homo sapiens GN=PROS1 PE=1 SV=1 - [PROS_HUMAN] | 102.11 | 37.87% | 26 |
| P62195 | 26S protease regulatory subunit 8 OS=Homo sapiens GN=PSMC5 PE=1 SV=1 - [PRS8_HUMAN] | 3.62 | 3.20% | 1 |
| O00231 | 26S proteasome non-ATPase regulatory subunit 11 OS=Homo sapiens GN=PSMD11 PE=1 SV=3 - [PSD11_HUMAN] | 0 | 2.61% | 1 |
| Q9UNM6 | 26S proteasome non-ATPase regulatory subunit 13 OS=Homo sapiens GN=PSMD13 PE=1 SV=2 - [PSD13_HUMAN] | 2.11 | 4.79% | 1 |
| Q99460 | 26S proteasome non-ATPase regulatory subunit 1 OS=Homo sapiens GN=PSMD1 PE=1 SV=2 - [PSMD1_HUMAN] | 9.43 | 5.46% | 4 |
| Q13200 | 26S proteasome non-ATPase regulatory subunit 2 OS=Homo sapiens GN=PSMD2 PE=1 SV=3 - [PSMD2_HUMAN] | 9.5 | 5.62% | 4 |
| O43242 | 26S proteasome non-ATPase regulatory subunit 3 OS=Homo sapiens GN=PSMD3 PE=1 SV=2 - [PSMD3_HUMAN] | 3.25 | 2.81% | 1 |
| Q13308 | Inactive tyrosine-protein kinase 7 OS=Homo sapiens GN=PTK7 PE=1 SV=2 - [PTK7_HUMAN] | 12.33 | 5.98% | 4 |
| P10586 | Receptor-type tyrosine-protein phosphatase F OS=Homo sapiens GN=PTPRF PE=1 SV=2 - [PTPRF_HUMAN] | 11.67 | 5.14% | 6 |
| Q13332 | Receptor-type tyrosine-protein phosphatase S OS=Homo sapiens GN=PTPRS PE=1 SV=3 - [PTPRS_HUMAN] | 12.56 | 3.85% | 4 |
| P53801 | Pituitary tumor-transforming gene 1 protein-interacting protein OS=Homo sapiens GN=PTTG1IP PE=1 SV=1 - [PTTG_HUMAN] | 2.87 | 6.67% | 1 |
| P31939 | Bifunctional purine biosynthesis protein PURH OS=Homo sapiens GN=ATIC PE=1 SV=3 - [PUR9_HUMAN] | 2.59 | 1.18% | 1 |
| Q15223 | Nectin-1 OS=Homo sapiens GN=PVRL1 PE=1 SV=3 - [PVRL1_HUMAN] | 2.04 | 2.71% | 1 |
| Q96NY8 | Nectin-4 OS=Homo sapiens GN=PVRL4 PE=1 SV=1 - [PVRL4_HUMAN] | 3.61 | 2.55% | 1 |
| P11216 | Glycogen phosphorylase, brain form OS=Homo sapiens GN=PYGB PE=1 SV=5 - [PYGB_HUMAN] | 7.31 | 4.03% | 1 |
| P06737 | Glycogen phosphorylase, liver form OS=Homo sapiens GN=PYGL PE=1 SV=4 - [PYGL_HUMAN] | 10.24 | 6.73% | 2 |
| P61026 | Ras-related protein Rab-10 OS=Homo sapiens GN=RAB10 PE=1 SV=1 - [RAB10_HUMAN] | 8.65 | 16.50% | 1 |
| P61106 | Ras-related protein Rab-14 OS=Homo sapiens GN=RAB14 PE=1 SV=4 - [RAB14_HUMAN] | 8.16 | 26.05% | 2 |
| Q15286 | Ras-related protein Rab-35 OS=Homo sapiens GN=RAB35 PE=1 SV=1 - [RAB35_HUMAN] | 7.77 | 16.42% | 1 |
| P51148 | Ras-related protein Rab-5C OS=Homo sapiens GN=RAB5C PE=1 SV=2 - [RAB5C_HUMAN] | 1.73 | 5.56% | 1 |
| Q9P0K7 | Ankycorbin OS=Homo sapiens GN=RAI14 PE=1 SV=2 - [RAI14_HUMAN] | 2.31 | 0.82% | 1 |
| Q8NFJ5 | Retinoic acid-induced protein 3 OS=Homo sapiens GN=GPRC5A PE=1 SV=2 - [RAI3_HUMAN] | 53.31 | 25.49% | 9 |
| P62826 | GTP-binding nuclear protein Ran OS=Homo sapiens GN=RAN PE=1 SV=3 - [RAN_HUMAN] | 14.87 | 23.61% | 6 |
| P61224 | Ras-related protein Rap-1b OS=Homo sapiens GN=RAP1B PE=1 SV=1 - [RAP1B_HUMAN] | 17.27 | 20.11% | 5 |
| P61225 | Ras-related protein Rap-2b OS=Homo sapiens GN=RAP2B PE=1 SV=1 - [RAP2B_HUMAN] | 5.42 | 12.57% | 2 |
| Q9H0H5 | Rac GTPase-activating protein 1 OS=Homo sapiens GN=RACGAP1 PE=1 SV=1 - [RGAP1_HUMAN] | 3.15 | 2.53% | 1 |
| P84095 | Rho-related GTP-binding protein RhoG OS=Homo sapiens GN=RHOG PE=1 SV=1 - [RHOG_HUMAN] | 2.02 | 9.95% | 1 |
| P62906 | 60S ribosomal protein L10a OS=Homo sapiens GN=RPL10A PE=1 SV=2 - [RL10A_HUMAN] | 2.56 | 5.99% | 1 |
| P26373 | 60S ribosomal protein L13 OS=Homo sapiens GN=RPL13 PE=1 SV=4 - [RL13_HUMAN] | 2.25 | 3.79% | 1 |
| P50914 | 60S ribosomal protein L14 OS=Homo sapiens GN=RPL14 PE=1 SV=4 - [RL14_HUMAN] | 2.52 | 5.12% | 1 |
| Q07020 | 60S ribosomal protein L18 OS=Homo sapiens GN=RPL18 PE=1 SV=2 - [RL18_HUMAN] | 4.41 | 6.91% | 1 |
| P84098 | 60S ribosomal protein L19 OS=Homo sapiens GN=RPL19 PE=1 SV=1 - [RL19_HUMAN] | 5.88 | 12.76% | 2 |
| P47914 | 60S ribosomal protein L29 OS=Homo sapiens GN=RPL29 PE=1 SV=2 - [RL29_HUMAN] | 3.75 | 9.43% | 1 |
| P42766 | 60S ribosomal protein L35 OS=Homo sapiens GN=RPL35 PE=1 SV=2 - [RL35_HUMAN] | 2.16 | 4.88% | 1 |
| P36578 | 60S ribosomal protein L4 OS=Homo sapiens GN=RPL4 PE=1 SV=5 - [RL4_HUMAN] | 2.33 | 2.11% | 1 |
| P46777 | 60S ribosomal protein L5 OS=Homo sapiens GN=RPL5 PE=1 SV=3 - [RL5_HUMAN] | 0 | 4.71% | 1 |
| P18124 | 60S ribosomal protein L7 OS=Homo sapiens GN=RPL7 PE=1 SV=1 - [RL7_HUMAN] | 6.13 | 12.10% | 2 |
| Q9H1E1 | Ribonuclease 7 OS=Homo sapiens GN=RNASE7 PE=1 SV=2 - [RNAS7_HUMAN] | 0 | 9.62% | 1 |
| P09651 | Heterogeneous nuclear ribonucleoprotein A1 OS=Homo sapiens GN=HNRNPA1 PE=1 SV=5 - [ROA1_HUMAN] | 2.09 | 4.84% | 1 |
| P62249 | 40S ribosomal protein S16 OS=Homo sapiens GN=RPS16 PE=1 SV=2 - [RS16_HUMAN] | 2.71 | 6.85% | 1 |
| P62851 | 40S ribosomal protein S25 OS=Homo sapiens GN=RPS25 PE=1 SV=1 - [RS25_HUMAN] | 2.76 | 7.20% | 1 |
| P62241 | 40S ribosomal protein S8 OS=Homo sapiens GN=RPS8 PE=1 SV=2 - [RS8_HUMAN] | 8.78 | 18.27% | 3 |
| Q9HCY8 | Protein S100-A14 OS=Homo sapiens GN=S100A14 PE=1 SV=1 - [S10AE_HUMAN] | 3.06 | 10.58% | 1 |
| Q96FQ6 | Protein S100-A16 OS=Homo sapiens GN=S100A16 PE=1 SV=1 - [S10AG_HUMAN] | 1.62 | 10.68% | 1 |
| P55011 | Solute carrier family 12 member 2 OS=Homo sapiens GN=SLC12A2 PE=1 SV=1 - [S12A2_HUMAN] | 11.76 | 4.21% | 3 |
| Q9Y666 | Solute carrier family 12 member 7 OS=Homo sapiens GN=SLC12A7 PE=1 SV=3 - [S12A7_HUMAN] | 3.29 | 1.02% | 1 |
| Q08357 | Sodium-dependent phosphate transporter 2 OS=Homo sapiens GN=SLC20A2 PE=1 SV=1 - [S20A2_HUMAN] | 17.42 | 9.36% | 5 |
| Q9UGH3 | Solute carrier family 23 member 2 OS=Homo sapiens GN=SLC23A2 PE=1 SV=1 - [S23A2_HUMAN] | 0 | 3.85% | 1 |
| Q9H2H9 | Sodium-coupled neutral amino acid transporter 1 OS=Homo sapiens GN=SLC38A1 PE=1 SV=1 - [S38A1_HUMAN] | 2.09 | 1.64% | 1 |
| Q96QD8 | Sodium-coupled neutral amino acid transporter 2 OS=Homo sapiens GN=SLC38A2 PE=1 SV=2 - [S38A2_HUMAN] | 13.87 | 12.45% | 4 |
| Q9ULF5 | Zinc transporter ZIP10 OS=Homo sapiens GN=SLC39A10 PE=1 SV=2 - [S39AA_HUMAN] | 1.67 | 1.20% | 1 |
| P23526 | Adenosylhomocysteinase OS=Homo sapiens GN=AHCY PE=1 SV=4 - [SAHH_HUMAN] | 20.14 | 18.52% | 8 |
| P53794 | Sodium/myo-inositol cotransporter OS=Homo sapiens GN=SLC5A3 PE=3 SV=2 - [SC5A3_HUMAN] | 4.33 | 2.65% | 2 |
| Q9Y289 | Sodium-dependent multivitamin transporter OS=Homo sapiens GN=SLC5A6 PE=2 SV=2 - [SC5A6_HUMAN] | 5.85 | 5.35% | 3 |
| P31641 | Sodium- and chloride-dependent taurine transporter OS=Homo sapiens GN=SLC6A6 PE=1 SV=2 - [SC6A6_HUMAN] | 11.17 | 5.65% | 3 |
| P48067 | Sodium- and chloride-dependent glycine transporter 1 OS=Homo sapiens GN=SLC6A9 PE=2 SV=3 - [SC6A9_HUMAN] | 1.79 | 1.27% | 1 |
| P31431 | Syndecan-4 OS=Homo sapiens GN=SDC4 PE=1 SV=2 - [SDC4_HUMAN] | 18.61 | 18.18% | 4 |
| O00560 | Syntenin-1 OS=Homo sapiens GN=SDCBP PE=1 SV=1 - [SDCB1_HUMAN] | 29.63 | 52.01% | 11 |
| O43175 | D-3-phosphoglycerate dehydrogenase OS=Homo sapiens GN=PHGDH PE=1 SV=4 - [SERA_HUMAN] | 3.44 | 2.81% | 1 |
| Q9NRX5 | Serine incorporator 1 OS=Homo sapiens GN=SERINC1 PE=1 SV=1 - [SERC1_HUMAN] | 3.52 | 3.75% | 1 |
| Q13530 | Serine incorporator 3 OS=Homo sapiens GN=SERINC3 PE=2 SV=2 - [SERC3_HUMAN] | 2.86 | 4.02% | 2 |
| Q86VE9 | Serine incorporator 5 OS=Homo sapiens GN=SERINC5 PE=2 SV=1 - [SERC5_HUMAN] | 8.64 | 9.93% | 3 |
| P50454 | Serpin H1 OS=Homo sapiens GN=SERPINH1 PE=1 SV=2 - [SERPH_HUMAN] | 2.78 | 2.63% | 1 |
| Q15393 | Splicing factor 3B subunit 3 OS=Homo sapiens GN=SF3B3 PE=1 SV=4 - [SF3B3_HUMAN] | 3.11 | 1.31% | 1 |
| Q99961 | Endophilin-A2 OS=Homo sapiens GN=SH3GL1 PE=1 SV=1 - [SH3G1_HUMAN] | 2.45 | 5.43% | 2 |
| O00161 | Synaptosomal-associated protein 23 OS=Homo sapiens GN=SNAP23 PE=1 SV=1 - [SNP23_HUMAN] | 0 | 6.64% | 1 |
| Q13103 | Secreted phosphoprotein 24 OS=Homo sapiens GN=SPP2 PE=1 SV=1 - [SPP24_HUMAN] | 3.34 | 5.69% | 1 |
| Q9Y5Y6 | Suppressor of tumorigenicity 14 protein OS=Homo sapiens GN=ST14 PE=1 SV=2 - [ST14_HUMAN] | 2.6 | 1.29% | 1 |
| Q658P3 | Metalloreductase STEAP3 OS=Homo sapiens GN=STEAP3 PE=1 SV=2 - [STEA3_HUMAN] | 2.55 | 2.25% | 1 |
| P27105 | Erythrocyte band 7 integral membrane protein OS=Homo sapiens GN=STOM PE=1 SV=3 - [STOM_HUMAN] | 15.75 | 22.57% | 5 |
| Q12846 | Syntaxin-4 OS=Homo sapiens GN=STX4 PE=1 SV=2 - [STX4_HUMAN] | 2.65 | 6.40% | 1 |
| P09758 | Tumor-associated calcium signal transducer 2 OS=Homo sapiens GN=TACSTD2 PE=1 SV=3 - [TACD2_HUMAN] | 32.98 | 34.67% | 8 |
| Q7L7X3 | Serine/threonine-protein kinase TAO1 OS=Homo sapiens GN=TAOK1 PE=1 SV=1 - [TAOK1_HUMAN] | 5.53 | 2.50% | 2 |
| P68363 | Tubulin alpha-1B chain OS=Homo sapiens GN=TUBA1B PE=1 SV=1 - [TBA1B_HUMAN] | 65.78 | 42.79% | 2 |
| P68366 | Tubulin alpha-4A chain OS=Homo sapiens GN=TUBA4A PE=1 SV=1 - [TBA4A_HUMAN] | 68.63 | 48.88% | 4 |
| Q9H4B7 | Tubulin beta-1 chain OS=Homo sapiens GN=TUBB1 PE=1 SV=1 - [TBB1_HUMAN] | 28.49 | 17.52% | 6 |
| P68371 | Tubulin beta-4B chain OS=Homo sapiens GN=TUBB4B PE=1 SV=1 - [TBB4B_HUMAN] | 44.46 | 24.27% | 2 |
| P07437 | Tubulin beta chain OS=Homo sapiens GN=TUBB PE=1 SV=2 - [TBB5_HUMAN] | 42.3 | 20.95% | 1 |
| P78371 | T-complex protein 1 subunit beta OS=Homo sapiens GN=CCT2 PE=1 SV=4 - [TCPB_HUMAN] | 10.12 | 7.66% | 3 |
| P48643 | T-complex protein 1 subunit epsilon OS=Homo sapiens GN=CCT5 PE=1 SV=1 - [TCPE_HUMAN] | 4.7 | 4.44% | 2 |
| Q99832 | T-complex protein 1 subunit eta OS=Homo sapiens GN=CCT7 PE=1 SV=2 - [TCPH_HUMAN] | 0 | 5.89% | 2 |
| P50990 | T-complex protein 1 subunit theta OS=Homo sapiens GN=CCT8 PE=1 SV=4 - [TCPQ_HUMAN] | 2.14 | 1.82% | 1 |
| Q15185 | Prostaglandin E synthase 3 OS=Homo sapiens GN=PTGES3 PE=1 SV=1 - [TEBP_HUMAN] | 2.69 | 10.63% | 1 |
| Q16473 | Putative tenascin-XA OS=Homo sapiens GN=TNXA PE=5 SV=2 - [TENXA_HUMAN] | 4.58 | 4.18% | 1 |
| P55072 | Transitional endoplasmic reticulum ATPase OS=Homo sapiens GN=VCP PE=1 SV=4 - [TERA_HUMAN] | 102.96 | 44.54% | 30 |
| P02786 | Transferrin receptor protein 1 OS=Homo sapiens GN=TFRC PE=1 SV=2 - [TFR1_HUMAN] | 53.97 | 27.37% | 17 |
| P05543 | Thyroxine-binding globulin OS=Homo sapiens GN=SERPINA7 PE=1 SV=2 - [THBG_HUMAN] | 5.03 | 4.58% | 2 |
| P00734 | Prothrombin OS=Homo sapiens GN=F2 PE=1 SV=2 - [THRB_HUMAN] | 116.09 | 27.97% | 20 |
| Q9Y490 | Talin-1 OS=Homo sapiens GN=TLN1 PE=1 SV=3 - [TLN1_HUMAN] | 18.26 | 2.83% | 6 |
| Q8NBN3 | Transmembrane protein 87A OS=Homo sapiens GN=TMEM87A PE=1 SV=3 - [TM87A_HUMAN] | 0 | 3.78% | 1 |
| Q9UHN6 | Transmembrane protein 2 OS=Homo sapiens GN=TMEM2 PE=1 SV=1 - [TMEM2_HUMAN] | 61.5 | 17.43% | 22 |
| Q9BSE2 | Transmembrane protein 79 OS=Homo sapiens GN=TMEM79 PE=1 SV=1 - [TMM79_HUMAN] | 2.8 | 3.05% | 1 |
| Q9HCN3 | Transmembrane protein 8A OS=Homo sapiens GN=TMEM8A PE=1 SV=3 - [TMM8A_HUMAN] | 32.7 | 10.64% | 7 |
| Q9H0E2 | Toll-interacting protein OS=Homo sapiens GN=TOLLIP PE=1 SV=1 - [TOLIP_HUMAN] | 3.68 | 5.11% | 1 |
| P11387 | DNA topoisomerase 1 OS=Homo sapiens GN=TOP1 PE=1 SV=2 - [TOP1_HUMAN] | 0 | 2.61% | 1 |
| P00750 | Tissue-type plasminogen activator OS=Homo sapiens GN=PLAT PE=1 SV=1 - [TPA_HUMAN] | 2.95 | 2.14% | 1 |
| Q13641 | Trophoblast glycoprotein OS=Homo sapiens GN=TPBG PE=1 SV=1 - [TPBG_HUMAN] | 3.85 | 7.14% | 2 |
| P60174 | Triosephosphate isomerase OS=Homo sapiens GN=TPI1 PE=1 SV=3 - [TPIS_HUMAN] | 6.63 | 8.74% | 2 |
| P29144 | Tripeptidyl-peptidase 2 OS=Homo sapiens GN=TPP2 PE=1 SV=4 - [TPP2_HUMAN] | 60.53 | 16.73% | 16 |
| P02787 | Serotransferrin OS=Homo sapiens GN=TF PE=1 SV=3 - [TRFE_HUMAN] | 38.41 | 25.21% | 17 |
| P02788 | Lactotransferrin OS=Homo sapiens GN=LTF PE=1 SV=6 - [TRFL_HUMAN] | 8 | 2.82% | 2 |
| Q8TD43 | Transient receptor potential cation channel subfamily M member 4 OS=Homo sapiens GN=TRPM4 PE=1 SV=1 - [TRPM4_HUMAN] | 3.08 | 1.24% | 1 |
| Q99816 | Tumor susceptibility gene 101 protein OS=Homo sapiens GN=TSG101 PE=1 SV=2 - [TS101_HUMAN] | 18.27 | 21.79% | 7 |
| Q63HK5 | Teashirt homolog 3 OS=Homo sapiens GN=TSHZ3 PE=1 SV=2 - [TSH3_HUMAN] | 2.21 | 2.31% | 1 |
| Q8NG11 | Tetraspanin-14 OS=Homo sapiens GN=TSPAN14 PE=1 SV=1 - [TSN14_HUMAN] | 8.89 | 15.93% | 3 |
| O14817 | Tetraspanin-4 OS=Homo sapiens GN=TSPAN4 PE=1 SV=1 - [TSN4_HUMAN] | 0 | 10.50% | 1 |
| O43657 | Tetraspanin-6 OS=Homo sapiens GN=TSPAN6 PE=1 SV=1 - [TSN6_HUMAN] | 3.15 | 4.90% | 1 |
| P07996 | Thrombospondin-1 OS=Homo sapiens GN=THBS1 PE=1 SV=2 - [TSP1_HUMAN] | 9.03 | 2.82% | 3 |
| Q9C0H2 | Protein tweety homolog 3 OS=Homo sapiens GN=TTYH3 PE=1 SV=3 - [TTYH3_HUMAN] | 22.92 | 7.27% | 4 |
| P0CG48 | Polyubiquitin-C OS=Homo sapiens GN=UBC PE=1 SV=3 - [UBC_HUMAN] | 131.35 | 86.72% | 11 |
| P06133 | UDP-glucuronosyltransferase 2B4 OS=Homo sapiens GN=UGT2B4 PE=1 SV=2 - [UD2B4_HUMAN] | 1.75 | 1.52% | 1 |
| Q03405 | Urokinase plasminogen activator surface receptor OS=Homo sapiens GN=PLAUR PE=1 SV=1 - [UPAR_HUMAN] | 10.14 | 14.33% | 3 |
| Q86UX7 | Fermitin family homolog 3 OS=Homo sapiens GN=FERMT3 PE=1 SV=1 - [URP2_HUMAN] | 9.27 | 4.80% | 3 |
| O60763 | General vesicular transport factor p115 OS=Homo sapiens GN=USO1 PE=1 SV=2 - [USO1_HUMAN] | 2.49 | 1.25% | 1 |
| P38606 | V-type proton ATPase catalytic subunit A OS=Homo sapiens GN=ATP6V1A PE=1 SV=2 - [VATA_HUMAN] | 3.5 | 2.43% | 1 |
| P18206 | Vinculin OS=Homo sapiens GN=VCL PE=1 SV=4 - [VINC_HUMAN] | 67.38 | 23.37% | 21 |
| Q9H9H4 | Vacuolar protein sorting-associated protein 37B OS=Homo sapiens GN=VPS37B PE=1 SV=1 - [VP37B_HUMAN] | 3.51 | 5.96% | 1 |
| Q93050 | V-type proton ATPase 116 kDa subunit a isoform 1 OS=Homo sapiens GN=ATP6V0A1 PE=1 SV=3 - [VPP1_HUMAN] | 10.5 | 7.29% | 4 |
| Q13488 | V-type proton ATPase 116 kDa subunit a isoform 3 OS=Homo sapiens GN=TCIRG1 PE=1 SV=3 - [VPP3_HUMAN] | 2.29 | 1.81% | 1 |
| Q9UN37 | Vacuolar protein sorting-associated protein 4A OS=Homo sapiens GN=VPS4A PE=1 SV=1 - [VPS4A_HUMAN] | 6.55 | 7.32% | 2 |
| P04004 | Vitronectin OS=Homo sapiens GN=VTN PE=1 SV=1 - [VTNC_HUMAN] | 79.05 | 32.43% | 15 |
| O75083 | WD repeat-containing protein 1 OS=Homo sapiens GN=WDR1 PE=1 SV=4 - [WDR1_HUMAN] | 8.28 | 14.52% | 5 |
| Q96DN5 | TBC1 domain family member 31 OS=Homo sapiens GN=TBC1D31 PE=2 SV=2 - [TBC31_HUMAN] | 0 | 2.53% | 1 |
| Q9UPY5 | Cystine/glutamate transporter OS=Homo sapiens GN=SLC7A11 PE=1 SV=1 - [XCT_HUMAN] | 11.53 | 13.77% | 6 |
| Q92536 | Y+L amino acid transporter 2 OS=Homo sapiens GN=SLC7A6 PE=1 SV=3 - [YLAT2_HUMAN] | 0 | 5.83% | 1 |
| Q9C0B5 | Palmitoyltransferase ZDHHC5 OS=Homo sapiens GN=ZDHHC5 PE=1 SV=2 - [ZDHC5_HUMAN] | 5.17 | 2.94% | 1 |
| Q07157 | Tight junction protein ZO-1 OS=Homo sapiens GN=TJP1 PE=1 SV=3 - [ZO1_HUMAN] | 13.28 | 7.95% | 8 |
| Q9UDY2 | Tight junction protein ZO-2 OS=Homo sapiens GN=TJP2 PE=1 SV=2 - [ZO2_HUMAN] | 5.62 | 2.94% | 2 |
| Q9UK55 | Protein Z-dependent protease inhibitor OS=Homo sapiens GN=SERPINA10 PE=1 SV=1 - [ZPI_HUMAN] | 3.06 | 2.25% | 1 |
| P23229 | Integrin alpha-6 OS=Homo sapiens GN=ITGA6 PE=1 SV=5 - [ITA6_HUMAN] | 140.84 | 37.26% | 35 |
